# Hybrid Immunity Protects against Antibody Fading after SARS-CoV-2mRNA Vaccination in Kidney Transplant Recipients, Dialysis Patients, and Medical Personnel: 9 Months Data from the Prospective, Observational Dia-Vacc Study

**DOI:** 10.3390/vaccines12070801

**Published:** 2024-07-19

**Authors:** Julian Stumpf, Torsten Siepmann, Jörg Schwöbel, Claudia Karger, Tom H. Lindner, Robert Faulhaber-Walter, Torsten Langer, Katja Escher, Kirsten Anding-Rost, Harald Seidel, Jan Hüther, Frank Pistrosch, Heike Martin, Jens Schewe, Thomas Stehr, Frank Meistring, Alexander Paliege, Daniel Schneider, Anne Steglich, Florian Gembardt, Friederike Kessel, Hannah Kröger, Patrick Arndt, Jan Sradnick, Kerstin Frank, Anna Klimova, René Mauer, Ingo Roeder, Torsten Tonn, Christian Hugo

**Affiliations:** 1 Department of Internal Medicine III, Division of Nephrology, University Hospital Carl Gustav Carus at the Technische Universität Dresden, Fetscherstraße 74, 01307 Dresden, Germany; julian.stumpf@ukdd.de (J.S.); alexander.paliege@ukdd.de (A.P.); daniel.schneider@ukdd.de (D.S.); anne.steglich2@ukdd.de (A.S.); florian.gembardt@ukdd.de (F.G.); friederike.kessel@tu-dresden.de (F.K.); hannah.weissbach@ukdd.de (H.K.); patrick.arndt@ukdd.de (P.A.); jan.sradnick@ukdd.de (J.S.); 2 KfH-Nierenzentrum Dresden, Fetscherstraße 73, 01307 Dresden, Germany; 3 KfH-Nierenzentrum am Klinikum Chemnitz, Krankenhaus Küchwald, Bürgerstraße 2, 09113 Chemnitz, Germany; torsten.siepmann@kfh.de; 4 Dialysezentrum Chemnitz, Forststraße 22, 09130 Chemnitz, Germany; joergschwoebel@dialysepraxis-chemnitz.de; 5 KfH-Nierenzentrum am Klinikum St. Georg, Delitzscher Straße 141, 04129 Leipzig, Germany; claudia.karger@kfh.de; 6 Division of Nephrology, University Hospital Leipzig, Liebigstraße 20, 04103 Leipzig, Germany; tom.lindner@medizin.uni-leipzig.de; 7 Nephrologisches Zentrum Freiberg, Franz-Kögler-Ring 135, 09599 Freiberg, Germany; r.faulhaber@dialyse-freiberg.de; 8 Dialysezentrum Annaberg, Geyersdorfer Hauptstraße 4, 09456 Annaberg-Buchholz, Germany; torsten.langer@t-online.de; 9 KfH-Gesundheitszentrum Aue, Albert-Schweitzer-Straße 33, 08280 Aue-Bad-Schlema, Germany; katja.escher@helios-gesundheit.de; 10 KfH-Nierenzentrum Bischofswerda, Kamenzer Straße 51, 01877 Bischofswerda, Germany; kirsten.anding-rost@kfh.de; 11 KfH-Nierenzentrum am Vogtland Krankenhaus Plauen, Röntgenstraße 6, 08529 Plauen, Germany; harald.seidel@kfh.de; 12 Nephrocare GmbH Döbeln, Grimmaische Straße 23, 04720 Döbeln, Germany; jan.huether@nephrocare.com; 13 Nephrologisches Zentrum Hoyerswerda, Liselotte-Herrmann-Straße 13, 02977 Hoyerswerda, Germany; dialyse-hoy@t-online.de; 14 Nephrologisches Zentrum Zwickau, Hilfegottesschachtstraße 3, 08056 Zwickau, Germany; h.martin@dialyse-zwickau.de; 15 Dialyse- und Nierenambulanz Sebnitz, Götzingerstraße 8, 01855 Sebnitz, Germany; dialyse_sebnitz@arcor.de; 16 KfH-Nierenzentrum Bautzen, Schäfferstraße 27, 02625 Bautzen, Germany; thomas.stehr@kfh.de; 17 KfH-Nierenzentrum am Städtischen Klinikum Görlitz, Girbigsdorfer Straße 26, 02828 Görlitz, Germany; frank.meistring@kfh.de; 18 Institut für Transfusionsmedizin Plauen, DRK-Blutspendedienst Nord-Ost Gemeinnützige GmbH, Röntgenstraße 2a, 08529 Plauen, Germany; k.frank@blutspende.de; 19 National Centre for Tumor Diseases (NCT) Partner Site Dresden, Fiedlerstraße 23, 01307 Dresden, Germany; anna.klimova@mailbox.tu-dresden.de; 20 Faculty of Medicine Carl Gustav Carus, Institute for Medical Informatics and Biometry (IMB), Technische Universität, Blasewitzer Straße 86, 01307 Dresden, Germany; rene.mauer@tu-dresden.de (R.M.); ingo.roeder@tu-dresden.de (I.R.); 21 Institute for Transfusion Medicine, German Red Cross Blood Donation Service North-East, Blasewitzer Straße 68/70, 01307 Dresden, Germany; t.tonn@blutspende.de; 22 Faculty of Medicine Carl Gustav Carus, Transfusion Medicine, Technische Universität, Fetscherstraße 74, 01307 Dresden, Germany

**Keywords:** COVID-19 vaccination, hybrid immunity, immunity fading, DiaVacc Study

## Abstract

(1) Background: Compared to medical personnel, SARS-CoV-2mRNA vaccination-related positive immunity rates, levels, and preservation over time in dialysis and kidney transplant patients are reduced. We hypothesized that COVID-19 pre-exposure influences both vaccination-dependent immunity development and preservation in a group-dependent manner. (2) Methods: We evaluated 2- and 9-month follow-up data in our observational Dia-Vacc study, exploring specific cellular (interferon-γ release assay = IGRA) and/or humoral immune responses (IgA/IgG/RBD antibodies) after two SARS-CoV-2mRNA vaccinations in 2630 participants, including medical personnel (301-MP), dialysis patients (1841-DP), and kidney transplant recipients (488-KTR). Study participants were also separated into COVID-19 pre-exposure (hybrid immunity) positive (n = 407) versus negative (n = 2223) groups. (3) Results: COVID-19 pre-exposure improved most vaccination-related positive immunity rates in KTR and DP at 2 months but not in MP, where rates reached almost 100% independent of hybrid immunity. In the COVID-19-negative study, patients’ immunity faded between two and nine months, evaluated via the percentage of patients with an RBD antibody decrease >50%, and was markedly group- (MP-17.8%, DP-52.2%, and KTR-38.6%) and vaccine type-dependent. In contrast, in all patient groups with COVID-19, pre-exposure RBD antibody decreases of >50% were similarly rare (MP-4.3%, DP-7.2%, and KTR-0%) but still vaccine type-dependent, with numerically reduced numbers in mRNA-1273- versus BNT162b2mRNA-treated patients. Multivariable regression analysis of RBD antibody changes between two and nine months by interval scale categorization confirmed COVID-19 pre-exposure as a factor in inhibiting strong RBD Ab fading. COVID-19 pre-exposure in MP and DP also numerically reduced T-cell immunity fading. In DP, symptomatic (versus asymptomatic) COVID-19 pre-exposure was identified as a factor in reducing strong RBD Ab fading after vaccination. (4) Conclusions: After mRNA vaccination, immunity positivity rates in DP and KTR but not MP, as well as immunity preservation in MP/DP/KTR, are markedly improved via prior COVID-19 infection. In DP, prior symptomatic compared to asymptomatic COVID-19 disease was particularly effective in blocking immunity fading after mRNA vaccination.

## 1. Introduction

In vulnerable groups such as dialysis and kidney transplant patients, SARS-CoV-2mRNA vaccination-related seroconversion and T-cell immune response quality and quantity, as well as its preservation over time, are reduced compared to a normal population such as medical personnel (MP) [1,2]. Due to COVID-19 disease, unprotected dialysis patients (DP) and kidney transplant recipients (KTR) experienced high mortality rates up to 20% [3,4]. Successful vaccination inducing specific humoral and/or cellular immunity can limit the severity of disease and is therefore especially important for immunocompromised or immunosuppressed high-risk populations [3,4,5]. Considering low mRNA vaccination-related seroconversion rates of 30–50% in KTR and the majority of DP patients as fast immunity faders after established humoral immunity, immune monitoring of these vulnerable groups may be considered an adequate tool to pilot individual patients through the pandemic and future outbreaks [1,2,6,7]. This concept of immune monitoring was realized in our multicenter, investigator-driven, prospective, observational Dia-Vacc study, which repetitively investigated a population of more than 3100 participants consisting of MP, DP, and KTR after vaccination with either BNT162b2mRNA or mRNA-1273. A substantial fraction of study participants were already exposed to the SARS-CoV-2 virus before vaccination could take place, which may influence both the development and preservation of the quality and quantity of the immune response after mRNA vaccination. Studies in normal populations investigating the interplay of immunity from natural COVID-19 infection and vaccination demonstrate that hybrid immunity offers the most robust protection against infection and severe disease; however, available evidence for immunocompromised or immunosuppressed patient populations is limited [8,9,10].

We hypothesized that COVID-19 pre-exposure-induced hybrid immunity influences not only the vaccination-dependent development of immunity but also protects against immunity fading depending on the vaccine type, patient group, and type of exposure (symptomatic versus asymptomatic).

## 2. Materials and Methods

### 2.1. Study Design

The non-interventional, investigator-driven, multicenter, prospective Dia-Vacc study (NCT number: 04799808) started with SARS-CoV-2 vaccination using either 2x BNT162b2mRNA or 2x 1273-mRNA in 26 nephrology centers from 15 January to 24 February 2021 and investigated the time course of a specific cellular and/or humoral immune response to disease and/or SARS-CoV-2 vaccination in MP, DP, and KTR [1]. From the complete Dia-Vacc study cohort, all participants exposed to two mRNA vaccinations (either 2x BNT162b2-mRNA or 2x mRNA-1273) between T0 and T2 (two months) were investigated and separated into a COVID group with COVID-19 pre-exposure, as found by positive PCR due to disease symptoms (symptomatic disease) or by anti-Spike or nucleocapsid antibodies before vaccination (asymptomatic disease). The non-COVID group without COVID-19 pre-exposure did not meet any of the criteria defined above. Immunity success rates after vaccination and dependent on COVID-19 pre-exposure were investigated in a Dia-Vacc cohort where de novo symptomatic or asymptomatic COVID-19 participants between T0 and T2 were excluded. For all immunity fading evaluations between T2 and T9 (nine months), de novo symptomatic or asymptomatic COVID-19 participants between T0 and T9, as well as participants with additional vaccinations between T2 and T9, were excluded.

SARS-CoV-2-specific IgG or IgA antibody reactions (anti-S1 IgA ratio ≥ 1.1 = positive result; anti-S1 IgG-binding antibody units/mL [BAU/mL] ≥ 35.2 = positive result) against subunit S1 of the Spike protein and IgG antibodies against the nucleocapsid protein subunit (NCP ratio ≥ 1.1 = positive result, synonymous with a history of COVID-19 infection) were analyzed in all study participants (eligibility if > 18 years old and signed informed consent) at T0 (vaccination start), T2, and T9 [8,11,12]. Receptor-binding domain (RBD, positive ≥ 35%) antibody formation, which suggests neutralizing activity against the SARS-CoV-2 virus, was additionally examined at T2 and T9. For all antibody measurements, Euroimmun ELISAs on Euroimmun analyzers were used and centrally measured [13]. The cellular SARS-CoV-2 immune response was examined using a SARS-CoV-2 specific interferon-γ release assay (IGRA ≥ 100 mIU/mL) at T0, T2, and T9 in representative subgroups. Further details on procedures and analysis have been described elsewhere [1,2].

**Figure 1:**
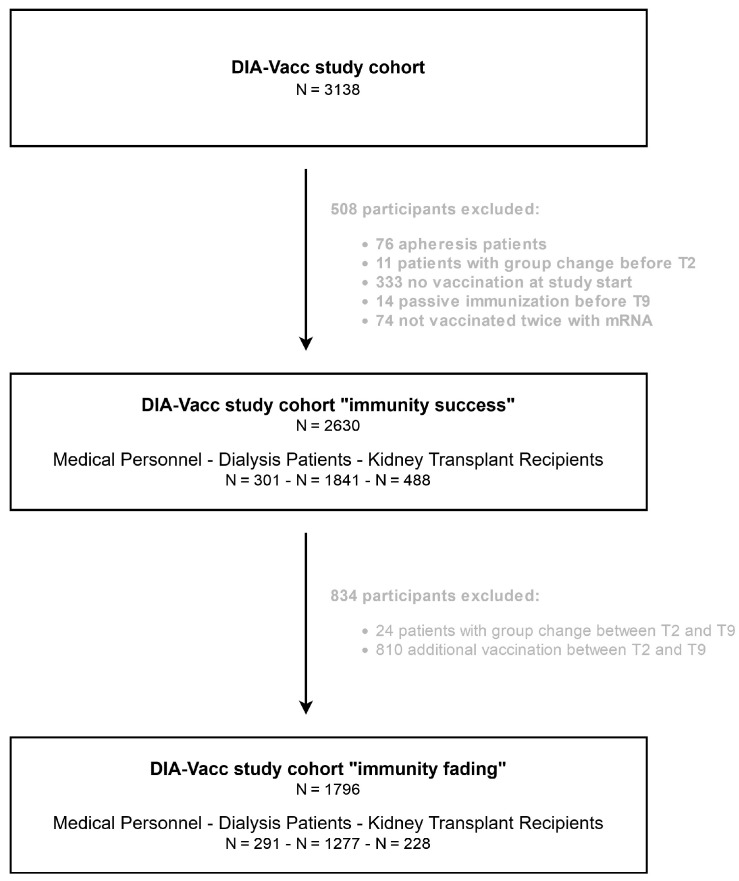
Study flow chart for the different study cohorts.

A study flow chart indicates the group changes and reasons for patient exclusions between T2 and T9 in the patient fraction observed regarding immunity fading (Figure 1).

### 2.2. End Points

The primary study end point is a positive humoral immune response after vaccination, as defined by positive IgG or IgA anti-SpikeS1 antibodies without de novo development of virus-specific NCP antibodies. Secondary end points were the development of vaccination-induced de novo T-cellular immunity and a clinical outcome, as well as serological and cellular immune response parameters and titers.

First, a 50% margin for (increased/equal/decreased) antibody and IGRA titer/value development was used to investigate the time course (T2 to T9) of an established vaccination-related immunity reaction for the different tests, and the percentage of patients within each margin was calculated for each group and each time point.

Second, the RBD time courses were analyzed on the interval scale, categorizing the detectable ranges of RBD antibody values into five intervals by labeling from 0 to 4 (referred to as “levels” in the data analysis) as described elsewhere [2]. For each patient, the change in levels between T2 and T9 was calculated. Strong antibody fading was defined as a downward change of more than two levels.

The high number of DP patients allowed the application of all evaluations into separated groups of asymptomatically and symptomatically diseased patients who had been pre-exposed to COVID-19.

### 2.3. Statistical Analysis

Baseline cohort characteristics and study end points, such as positive immunity rates, immunity titers, and immunity fading rates, were summarized as absolute frequencies and percentages, mean and standard deviation, or median and interquartile range (IQR), as appropriate. The differences in group rates and proportions were assessed using the chi-square test. The Mann–Whitney U test or Wilcoxon signed-rank test was applied to compare the medians between or within the study subgroups, depending on the context.

Multivariable regression analysis was performed to assess the time effects and group effects in RBD responses. The potential risk factors of patients with a strong antibody decline were investigated using logistic regression. The vaccine type was included among the risk factors due to the possible occurrence of a substantial difference in seroconversion and fading response after administering different vaccines in each logistic regression model, as was observed in a number of studies [7,8]. Other potential risk factors, common to all study groups, were age, gender, diabetes mellitus diagnosis, and body mass index (BMI). A logistic regression model was fitted to each study group separately and to all groups jointly. Adjusting for age aimed to reduce of the confounding effect due to heterogeneity among age distributions in the study groups. In addition to the risk factors listed above, the models fitted for DP and KTR separately contained the effects of hepatitis B vaccination failure, as well as group-specific effects: time on dialysis (DP) and time after transplantation (KTR). In addition, the number of immunosuppressive drugs was used as a covariate in the model for KTR, and the indicator of the presence of IS was included in the model for DP. A logistic regression model fitted for all groups jointly included the group effect in addition to other common risk factors.

For hypothesis testing, the significance level of 5% (two-sided) was chosen. A Bonferroni correction was applied during post-hoc testing of group effects. Data analysis was implemented in the R Environment for Statistical Computing, Version 4.0.4. R, the script of which can be provided upon request [11].

## 3. Results

### 3.1. Basic Study Cohort Characteristics (Table 1 and Table 2)

Basic patient characteristics of the subgroups regarding vaccination-related immunity rates at T2 are shown in Table 1. Data regarding immunity fading between T2 and T9 are shown in Table 2, where a significant number of additional study participants (especially KTR) had to be excluded due to additional vaccinations. Hereby, a total of 2630/1796 study participants, of which 407/314 had pre-exposure to COVID-19 before vaccination, qualified for immunity rate and titer (at T2) or immunity fading (between T2 and 9) evaluations, respectively. Study characteristics are quite similar within subgroups with/without COVID-19 pre-exposure. However, when different subgroups are compared (MP vs. DP vs. KTR, Table 1 and Table 2), the characteristics are different for several variables, such as age, sex, comorbidities, immunosuppressive drug exposure, type of vaccine, and type of COVID-19 pre-exposure.

**Table 1:** Baseline study cohort characteristics for vaccination-related immunity success at T2 (two months) of different DIA-Vacc study groups (MP, DP, and KTR), separated by hybrid immunity (-COV) or its absence (-NoCOV).

*Variable*	*Category*	*MP-NoCOV*	*MP-COV*	*DP-NoCOV*	*DP-COV*	*KTR-NoCOV*	*KTR-COV*
***Number (n/%)***	**evaluable**	240/100	61/100	1531/100	310/100	452/100	36/100
***Age (years)***	**mean ± SD**	47.2 ± 11.5	48.1 ± 10.3	67.4 ± 13.9	69.4 ± 12.4	57.2 ± 13.5	60.2 ± 9.9
***Male Sex***	**n/%**	54/22.5	6/10	1006/65.8	198/64.3	292/64.6	24/66.7
***BMI (kg/m^2^)***	**mean ± SD**	26 ± 5.1	25.1 ± 4.6	27.5 ± 5.7	28.1 ± 6.1	26.2 ± 4.7	26.7 ± 4.3
***Cause of end-stage renal disease***	**n/%**	n.a.	n.a.	1262/82.4	255/82.3	280/61.9	25/69.4
Diabetes/Hypertension/Vascular disease	n/%	n.a.	n.a.	744/48.6	158/51	78/17.3	10/27.8
Glomerulonephritis/Interstitial nephritis	n/%	n.a.	n.a.	334/21.8	75/24.2	125/27.7	10/27.8
Vasculitis	n/%	n.a.	n.a.	54/3.5	3/1	12/2.7	0/0
Polycystic kidney disease	n/%	n.a.	n.a.	130/8.5	19/6.1	65/14.4	5/13.9
Unknown	n/%	n.a.	n.a.	269/17.6	55/17.7	172/38.1	11/30.6
***Drug-treated comorbidities***	**n/%**	56/23.3	11/18	1473/96.2	303/97.7	405/89.6	32/88.9
Chronic kidney disease	n/%	3/1.2	0/0	1531/100	310/100	452/100	36/100
Diabetes mellitus	n/%	7/2.9	2/3.3	523/34.2	153/49.4	80/17.7	6/16.7
Cardiovascular disease	n/%	44/18.3	6/9.8	1424/93	297/95.8	390/86.3	32/88.9
Lung disease	n/%	8/3.3	4/6.6	101/6.6	27/8.7	28/6.2	2/5.6
Liver cirrhosis	n/%	0/0	0/0	22/1.4	3/1	3/0.7	0/0
Cancer	n/%	2/0.8	1/1.6	80/5.2	24/7.7	14/3.1	2/5.6
None	n/%	184/76.7	50/82	58/3.8	7/2.3	47/10.4	4/11.1
***Type of dialysis***		n.a.	n.a.	1519/99.2	308/99.4	n.a.	n.a.
Hemodialysis	n/%	n.a.	n.a.	1477/97.2	303/98.4	n.a.	n.a.
Peritoneal dialysis	n/%	n.a.	n.a.	42/2.8	5/1.6	n.a.	n.a.
***Time on dialysis (years)***	***mean ± SD***	n.a.	n.a.	5.7 ± 5.6	6 ± 6	6.3 ± 6.6	6.2 ± 4.6
***On transplant waiting list***	***n/%***	n.a.	n.a.	217/14.2	36/11.6	n.a.	n.a.
***Time on transplantation (years)***	***mean ± SD***	n.a.	n.a.	n.a.	n.a.	9.9 ± 7	10 ± 6.6
***Previous transplantation***	**n/%**	n.a.	n.a.	116/7.6	21/6.8	73/16.2	6/16.7
***Hepatitis B vaccination failure***	**n/%**	4/1.7	4/6.6	305/19.9	62/20	47/10.4	5/13.9
***Flu vaccination winter 2020/21***	**n/%**	145/60.4	38/62.3	1125/73.5	214/69	255/56.4	22/61.1
***On immunosuppressive therapy***	**n/%**	3/1.2	1/1.6	83/5.4	11/3.5	446/98.7	36/100
Corticosteroids	n/%	2/0.8	1/1.6	54/3.5	4/1.3	214/47.3	14/38.9
Calcineurin Inhibitor	n/%	0/0	0/0	24/1.6	6/1.9	394/87.2	32/88.9
MMF/MPA	n/%	0/0	0/0	17/1.1	1/0.3	345/76.3	29/80.6
mTOR Inhibitor	n/%	0/0	0/0	3/0.2	0/0	70/15.5	3/8.3
Belatacept	n/%	0/0	0/0	2/0.1	0/0	18/4	1/2.8
T-cell depleting ab	n/%	0/0	0/0	0/0	0/0	0/0	0/0
B-cell depleting ab	n/%	2/0.8	0/0	7/0.5	0/0	1/0.2	0/0
Other	n/%	1/0.4	0/0	6/0.4	1/0.3	9/2	0/0
***Type of vaccine***							
BNT162b2 mRNA	n/%	96/40	18/29.5	328/21.4	39/12.6	143/31.6	10/27.8
mRNA-1273	n/%	144/60	43/70.5	1203/78.6	271/87.4	309/68.4	26/72.2
***COVID-19 disease at/before T0***	**n/%**	**0/0**	**61/100**	**0/0**	**310/100**	**0/0**	**36/100**
Asymptomatic COVID-19	n/%	0/0	17/27.9	0/0	156/50.3	0/0	24/66.7
Symptomatic COVID-19	n/%	0/0	44/72.1	0/0	154/49.7	0/0	12/33.3

**Table 2:** Baseline study cohort characteristics for immunity fading between T2 (two months) and T9 (nine months) of different DIA-Vacc study groups (MP, DP, and KTR), separated by hybrid immunity (-COV) or its absence (-NoCOV).

*Variable*	*Category*	*MP-NoCOV*	*MP-COV*	*DP-NoCOV*	*DP-COV*	*KTR-NoCOV*	*KTR-COV*
***Number (n/%)***	**evaluable**	231/100	60/100	1049/100	228/100	202/100	26/100
***Age (years)***	**mean ± SD**	46.8 ± 11.4	47.9 ± 10.3	66.8 ± 14.3	68.8 ± 13	55.4 ± 13.5	58.8 ± 10.5
***Male Sex***	**n/%**	50/21.6	6/10.2	690/66	144/63.7	129/63.9	16/61.5
***BMI (kg/m^2^)***	**mean ± SD**	25.8 ± 5.1	25.2 ± 4.7	27.5 ± 5.7	27.5 ± 5.6	26.4 ± 4.7	26.3 ± 4.3
***Cause of end-stage renal disease***	**n/%**	n.a.	n.a.	858/81.8	184/80.7	125/61.9	18/69.2
Diabetes/Hypertension/Vascular disease	n/%	n.a.	n.a.	506/48.2	109/47.8	36/17.8	5/19.2
Glomerulonephritis/Interstitial nephritis	n/%	n.a.	n.a.	218/20.8	56/24.6	52/25.7	10/38.5
Vasculitis	n/%	n.a.	n.a.	40/3.8	3/1.3	5/2.5	0/0
Polycystic kidney disease	n/%	n.a.	n.a.	94/9	16/7	32/15.8	3/11.5
Unknown	n/%	n.a.	n.a.	191/18.2	44/19.3	77/38.1	8/30.8
***Drug-treated comorbidities***	**n/%**	52/22.5	10/16.7	1002/95.5	221/96.9	180/89.1	22/84.6
Chronic kidney disease	n/%	1/0.4	0/0	1049/100	228/100	202/100	26/100
Diabetes mellitus	n/%	7/3	1/1.7	339/32.3	105/46.1	32/15.8	4/15.4
Cardiovascular disease	n/%	40/17.3	6/10	964/91.9	217/95.2	172/85.1	22/84.6
Lung disease	n/%	8/3.5	4/6.7	71/6.8	19/8.3	15/7.4	2/7.7
Liver cirrhosis	n/%	0/0	0/0	16/1.5	3/1.3	0/0	0/0
Cancer	n/%	1/0.4	1/1.7	57/5.4	20/8.8	9/4.5	1/3.8
None	n/%	179/77.5	50/83.3	47/4.5	7/3.1	22/10.9	4/15.4
***Type of dialysis***		n.a.	n.a.	1049/100	228/100	n.a.	n.a.
Hemodialysis	n/%	n.a.	n.a.	1021/97.3	224/98.2	n.a.	n.a.
Peritoneal dialysis	n/%	n.a.	n.a.	28/2.7	4/1.8	n.a.	n.a.
***Time on dialysis (years)***	***mean ± SD***	n.a.	n.a.	5.9 ± 5.7	6.1 ± 6	6.4 ± 6.8	6.2 ± 3.9
***On transplant waiting list***	***n/%***	n.a.	n.a.	161/15.3	30/13.2	n.a.	n.a.
***Time on transplantation (years)***	***mean ± SD***	n.a.	n.a.	n.a.	n.a.	10.8 ± 7.9	10.1 ± 6.7
***Previous transplantation***	**n/%**	n.a.	n.a.	93/8.9	19/8.3	39/19.3	3/11.5
***Hepatitis B vaccination failure***	**n/%**	4/1.7	4/6.7	208/19.8	42/18.4	22/10.9	5/19.2
***Flu vaccination winter 2020/21***	**n/%**	137/59.3	37/61.7	784/74.7	157/68.9	108/53.5	16/61.5
***On immunosuppressive therapy***	**n/%**	2/0.9	1/1.7	61/5.8	10/4.4	197/97.5	26/100
Corticosteroids	n/%	1/0.4	1/1.7	41/3.9	3/1.3	100/49.5	10/38.5
Calcineurin Inhibitor	n/%	0/0	0/0	20/1.9	6/2.6	165/81.7	24/92.3
MMF/MPA	n/%	0/0	0/0	12/1.1	1/0.4	126/62.4	20/76.9
mTOR Inhibitor	n/%	0/0	0/0	2/0.2	0/0	45/22.3	2/7.7
Belatacept	n/%	0/0	0/0	1/0.1	0/0	5/2.5	1/3.8
T-cell depleting ab	n/%	0/0	0/0	0/0	0/0	0/0	0/0
B-cell depleting ab	n/%	1/0.4	0/0	3/0.3	0/0	0/0	0/0
Other	n/%	1/0.4	0/0	5/0.5	1/0.4	7/3.5	0/0
***Type of vaccine***							
BNT162b2 mRNA	n/%	89/38.5	17/28.3	177/16.9	29/12.7	41/20.3	7/26.9
mRNA-1273	n/%	142/61.5	43/71.7	872/83.1	199/87.3	161/79.7	19/73.1
***COVID-19 disease at/before T0***	**n/%**	**0/0**	**60/100**	**0/0**	**228/100**	**0/0**	**26/100**
Asymptomatic COVID-19	n/%	0/0	17/28.3	0/0	118/51.8	0/0	16/61.5
Symptomatic COVID-19	n/%	0/0	43/71.7	0/0	110/48.2	0/0	10/38.5

### 3.2. Study End Points

Positive immunity rates and titers at two months (T2) after vaccination are dependent on COVID-19 pre-exposure in DP and KTR but less so in MP.

In MP, humoral responses as assessed by general seroconversion (as defined by positive IgA or IgG antibodies) and positivity rates for IgA/IgG/RBD antibodies, as well as assay titers, were best/highest and largely independent of COVID pre-exposure (Table 3). In DP, vaccination-dependent seroconversion/antibody rates and titers were already high without pre-exposure but increased slightly after COVID pre-exposure (Table 3). In KTR, COVID pre-exposure improved the otherwise low immunity responses after vaccination to almost MP/DP levels by almost doubling antibody positivity rates and markedly elevating antibody titers against the SpikeS1 protein (Table 3). Very similar results/trends were seen regarding cellular immunity responses (IGRA) with the exception of KTR, where the number of IGRA measurements in patients pre-exposed to COVID-19 was not sufficient for comparison (Table 3).

Vaccine type did not play a substantial role in antibody positivity rates or titers with/without COVID-19 pre-exposure in the MP group (Table S1A). As reported before for DP and KTR without previous COVID-19 exposure, seroconversion/antibody positivity rates and titers were higher in mRNA1273-treated patients compared to BNT162b2 mRNA patients (Table S1B). In DP as well as in KTR, these vaccine-dependent differences largely vanished (with the exception of IgA-ab against the Spike protein) and reached uniformly excellent results when COVID-19 pre-exposed patients were also vaccinated (Table S1B).

Multivariable analysis of all study participants demonstrates that besides the MP group, vaccine type mRNA-1273, younger age, and especially COVID-19 pre-exposure is a strong success/“risk” factor for RBD antibody seropositivity (Table 4). Performing this analysis in the different patient subgroups also shows that COVID-19 pre-exposure is a success factor for vaccination-dependent RBD seropositivity in DP and KTR but not in MP (Table 4).

Table 1, Table 3 and Table 4 describes the data relating to 2630 DiaVacc study participants (MP, DP, and KTR) who were evaluated for vaccination-dependent humoral or cellular positivity after two vaccinations at the two-month time point (T2). The patients were separated by hybrid immunity due to COVID-19 infection prior to vaccination (-COV) or its absence (-NoCOV).

Table 1 describes the baseline study cohort characteristics. Positive immunity rates and titers (Table 3) are demonstrated for all of the different subgroups vaccinated with either 2x BNT162b2 mRNA or 2x mRNA-1273. Table 4 shows “risk” factors for RBD antibody positivity (>35%) in all participants, medical personnel (MP), dialysis patients (DP), or kidney transplant recipients (KTR) by multivariable analysis.

Participants experiencing asymptomatic* or documented symptomatic** COVID-19 disease beyond T0 (also T1–T9) were excluded from the immune response assessment in all tables.

Humoral immunity fading between two and nine months after vaccination is markedly attenuated by COVID-19 pre-exposure and is dependent on the type of pre-exposure and mRNA vaccine.

**Figure 2:**
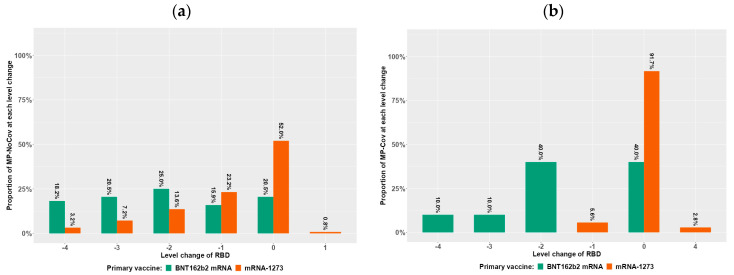

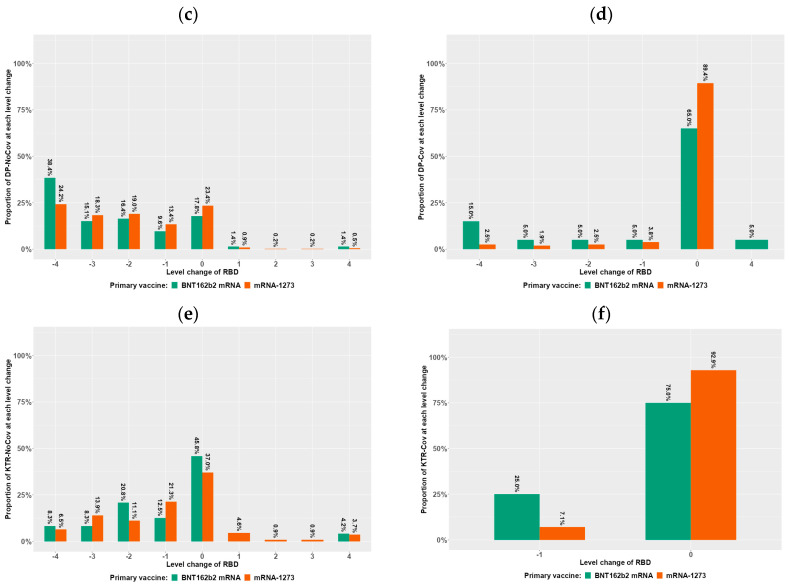
The change of RBD level categorization between T2 and T9 for different patient groups dependent on hybrid immunity (and vaccine type); (**a**) MP without hybrid immunity; (**b**) MP with hybrid immunity; (**c**) DP without hybrid immunity; (**d**) DP with hybrid immunity; (**e**) KTR without hybrid immunity; (**f**) KTR with hybrid immunity.

Similar to successful seroconversion after vaccination, immunity fading between T2 and T9 was differentially influenced by COVID-19 pre-exposure (Figure 2, Table 2, Table 5, Table 6 and Table S2). In MP, immunity fading (as indicated by a 50% titer decrease within the time period) considering IgG or RBD antibodies was the lowest compared to other subgroups such as DP/KTR, but was still attenuated by COVID-19 pre-exposure (Table 5 and Table S2A–C). Interestingly, IgA antibody fading in MP was as severe as in other subgroups and was not markedly influenced by COVID pre-exposure when compared to DP/KTR (Table 5 and Table S2A–C). In DP, vaccination-dependent IgA/IgG/RBD antibody fading was highest in patients without COVID-19 but markedly attenuated by COVID-19 pre-exposure (Table 5 and Table S2A–C). In KTR, IgA/IgG/RBD antibody fading in patients without COVID-19 pre-exposure was intermediate and stopped with COVID-19 pre-exposure. Similar trends were seen regarding cellular immunity responses (IGRA), with the exception of KTR, where the number of IGRA measurements in patients pre-exposed to COVID-19 was not sufficient for comparison.

Figure 2 describes the change of RBD level categorization between T2 and T9 as a measure for antibody fading for different patient groups (MD, DP, and KTR) dependent on hybrid immunity and vaccine type.

In the proposed interval classification, Level 0 is assigned to RBD values below the corresponding positivity threshold (35% inhibition [IH]), and the remaining values are divided into four intervals of approximately equal length (Level 1 = 35 up to < 50% IH; Level 2 = 50 up to < 65% IH; Level 3 = 65 up to < 80% IH; Level 4 = ≥ 80% IH). This figure depicts the quantitative changes between T2 and T9, which are dependent on patient group (MP = Medical Personnel in Figure 2a,b, DP = Dialysis Patients in Figure 2c,d, and KTR = Kidney Transplant Recipients in Figure 2e,f) and primary vaccine type (green represents BNT162b2mRNA and red represents 1273-mRNA), as well as on whether hybrid immunity due to COVID-19 infection prior to vaccination was present (panels Figure 2b,d,f) or absent (panels Figure 2a,c,e). These intervals can be used to quantify the change in RBD categories between T2 and T9, where, for example, a negative change corresponds to a decrease in RBD, respectively, with a change of 4 being the maximum decrease. Based on this definition, we referred to any decrease of more than two levels (at least three) as a “strong antibody decline”. Participants experiencing asymptomatic* or documented symptomatic** COVID-19 disease beyond T0 (also T1–T9) were excluded from the immune response assessment in all figures.

With the exception of IgA antibodies, the vaccine type plays a substantial role in IgG and RBD antibody fading in all subgroups, where mRNA-1273 better prevents accelerated fading compared to BNT162b2-mRNA (Table 5 and Table S2A–C). This influence seems to be conserved in patients with COVID-19 pre-exposure, leading to an almost complete inhibition of fading with mRNA-1273 (Table 5 and Table S2A–C). In contrast, IgA antibody fading in all subgroups did not seem to be altered by vaccine type (Table 5 and Table S2A–C). Similar trends also seem to be relevant for cellular immunity monitoring, but measurement numbers for IGRAs should be higher before vaccine types can be compared.

Multivariable logistic regression was used to investigate the potential risk factors for strong RBD antibody decline between T2 and T9 (Table 6 and Table S3). The analysis applied to all study participants identified the DP group, vaccine type BNT162b2-mRNA, and older age as risk factors for strong RBD-antibody decline, while COVID-19 pre-exposure strongly prevented it (Table 6). Performing this analysis in the different patient subgroups also showed that COVID-19 pre-exposure had a preventative effect on vaccination-dependent RBD-antibody fading in MP and DP (Table 6). Post-hoc pairwise testing showed that the strong decline in RBD antibodies was about three/five times less likely for MP/DP participants with COVID-19 pre-exposure. The number of patients, especially in the COVID-19 pre-exposure group, was not large enough for a similar analysis in the KTR group (Table 6).

Table 2, Table 5 and Table 6 describes the data relating to 1796 DiaVacc study participants (MP, DP, and KTR), who were evaluated for vaccination-dependent humoral or cellular positivity after two vaccinations at the nine-month mark (T9) in comparison to the two-month time point (T2). The participants were separated by the presence of hybrid immunity due to prior COVID-19 infection (-COV) or its absence (-NoCOV), with a focus on immunity fading.

Table 2 describes the baseline study cohort characteristics for the immune fading evaluations. The extent of immunity fading was calculated for T9 in comparison with T2 for all different subgroups together or separately in participants being vaccinated with either 2x BNT162b2 mRNA or 2x mRNA-1273. To assess immunity fading of antibody or IGRA titers between T2 and T9, a 50% margin was categorized as decreased (<50%). Table 6 shows the risk factors for RBD antibody fading between two and nine months after the start of vaccination in all participants, medical personnel (MP), dialysis patients (DP), or kidney transplant recipients (KTR), by multivariable analysis. Hereby, RBD level categorization changes between T2 and T9 were used as a measure for RBD antibody fading for multivariable analysis, as described in the methods. Participants experiencing asymptomatic* or documented symptomatic** COVID-19 disease beyond T0 were excluded from the immune response assessment in all tables.

Using RBD level categorization changes between T2 and T9 as a measure for RBD antibody fading/decline separated by COVID-19 pre-exposure/hybrid immunity (Figure 2b,d,f and Figure 2a,c,e) and mRNA vaccine type (BNT162b2-mRNA in green and mRNA-1273 in brown), the above results from the multivariable analysis can be appreciated visually (Figure 2). Negative level changes from −1 to −4 levels expressing the degree of RBD antibody fading are frequent in all study subgroups without COVID-19 pre-exposure and less frequent in subgroups with hybrid immunity. Hereby, study participants who were pre-exposed to COVID-19 and vaccinated with mRNA-1273 demonstrated hardly any or only minor level changes between two and nine months (Figure 2b,d,f).

### 3.3. The Type of Clinical COVID-19 Disease Pre-Exposure (Symptomatic versus Asymptomatic) Is Important for Preservation of Hybrid Immunity in Dialysis Patients

To further explore the question of whether the type of clinical COVID-19 pre-exposure and/or preservation of hybrid immunity plays a role in development, due to its size and the COVID-19-dependent inhibition of a strong immunity decline in NoCOV groups, the DP-COV group was used.

After separating the pre-exposed COVID DP group into asymptomatic versus symptomatic PCR-positive COVID-19 subgroups, no differences can be found regarding vaccination-dependent positive immunity rates at T2, since overall antibody positivity rates were close to 100% and positive T-cell immunity rates were around 86% (Table 3). In addition, all antibody and IGRA titers were in a very high range (Table 3). Using multivariable analysis, the development of RBD-Ab positivity at T2 was not dependent on the clinical substatus of COVID-19 (Table 7).

Table 7 shows success factors for RBD antibody positivity at T2.

Participants experiencing asymptomatic* or documented symptomatic** COVID-19 disease beyond T0 were excluded from the immune response assessment.

The definition of hepatitis B vaccination failure is patients with unsuccessful vaccination after at least four attempts.

**Figure 3:**
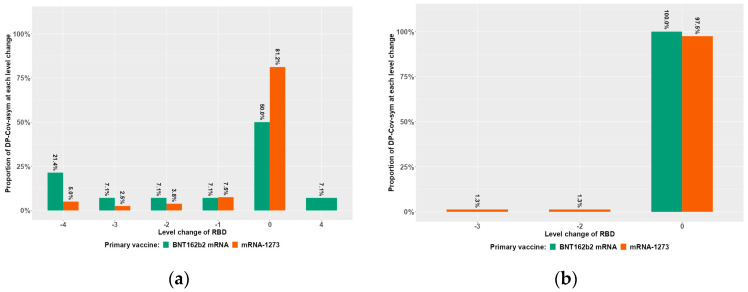
The change of RBD level categorization between T2 and T9 in dialysis patients (DP) dependent on the clinical type of hybrid immunity (asymptomatic versus symptomatic); (**a**) DP with asymptomatic hybrid immunity; (**b**) DP with symptomatic hybrid immunity.

In contrast, using an RBD level categorization change of at least three levels between T2 and T9 as a measure for “strong RBD antibody decline”, separated by COVID-19 pre-exposure type, multivariable analysis indicated symptomatic disease as an independent factor, being eight times more inhibitory of a strong RBD-Ab decline compared to asymptomatic disease (Table 8). These results can also be appreciated visually, where RBD level categorization changes are shown in a clinical subtype- (Figure 3) and mRNA vaccine type (BNT162b2-mRNA in green and mRNA-1273 in brown)-dependent manner. Supporting RBD level categorization change data, humoral immunity fading, as indicated by a 50% titer decrease within the time period, occurred in reduced rates in symptomatically versus asymptomatically pre-exposed DP, representing 16% versus 34% (*p* = 0.012) for IgA-Ab, 1% versus 15% for IgG-Ab (*p* = 0.003), and 1% versus 13% for RBD-Ab (*p* = 0.007) (data not in tables).

Table 8 shows risk factors for strong RBD-Ab fading between two (T2) and nine months (T9) after the start of vaccination.

Participants experiencing asymptomatic* or documented symptomatic** COVID-19 disease beyond T0 were excluded from the immune response assessment.

The definition of hepatitis B vaccination failure is patients with unsuccessful vaccination after at least four attempts.

Figure 3 describes the change of RBD level categorization between T2 and T9 as a measure for antibody fading for dialysis patients (DP) dependent on the clinical type of hybrid immunity (and vaccine type).

In the proposed interval classification, Level 0 is assigned to RBD values below the corresponding positivity threshold (35% inhibition [IH]), and the remaining values are divided into four intervals of approximately equal length (Level 1 = 35 up to < 50% IH; Level 2 = 50 up to < 65% IH; Level 3 = 65 up to < 80% IH; Level 4 =≥ 80% IH). This figure depicts the quantitative changes between T2 and T9, which are dependent on the clinical type of hybrid immunity (asymptomatic COVID-19 infection prior to vaccination (Figure 3a) versus symptomatic COVID-19 infection prior to vaccination (Figure 3b)) in DP, as demonstrated in a primary vaccine type-dependent manner (green represents BNT162b2mRNA and red represents 1273-mRNA). These intervals can be used to quantify the change in RBD categories between T2 and T9, where, for example, a negative change corresponds to a decrease in RBD, respectively, with a change of 4 being the maximum decrease. Based on this definition, we referred to any decrease of more than two levels (at least three) as a “strong antibody decline”. Participants experiencing asymptomatic* or documented symptomatic** COVID-19 disease beyond T0 (also T1–T9) were excluded from the immune response assessment in all figures.

### 3.4. Clinical Outcome (Data Provided in Text Below)

Side effects at first vaccination were numerically more frequent in all groups with COVID-19 pre-exposure compared to those without pre-exposure (34% vs. 26% in MP; 20% vs. 12% in DP; 50% vs. 33% in KTR, respectively). This difference was almost equalized at the second vaccination (43% vs. 40% in MP; 28% vs. 24% in DP; 37% vs. 35% in KTR, respectively). Between T0 and T9, MP without COVID-19 pre-exposure experienced de novo asymptomatic/symptomatic COVID-19 disease at a frequency of 0.4%/1.3% and 4.9%/1.6% with pre-exposure, respectively. The DP-NoCOV group had a frequency of 1.4%/2.2% and the DP-COV group had a frequency of 10.6%/1.0% for de novo asymptomatic/symptomatic COVID-19 disease, respectively. The KTR-NoCOV group showed a frequency of 0.4%/1.8% and the KTR-COV group showed a frequency of 8.3%/2.8% for de novo asymptomatic/symptomatic COVID-19 disease, respectively. Therefore, the relationship between asymptomatic and symptomatic COVID-19 disease was markedly increased (from seven to twenty times) in COV groups. Between T2 and T9, hospitalization due to de novo COVID-19 was only necessary in 0.3% of DP-NoCOV, 0.6% of KTR-NoCov, and 0% in all other groups. Intensive care treatment or death occurred only in 0.1% of the DP-NoCOV group.

## 4. Discussion

Our study demonstrates that positive immunity rates and titers at two months after the start of vaccination are dependent on COVID-19 pre-exposure/hybrid immunity in DP and KTR but less so in MP. In MP, vaccination-related humoral and cellular immunity rates close to 100% and high titers after two months are practically too good to be improved. In contrast, COVID-19 pre-exposure markedly improves vaccine-related immunity rates and titers in the DP population to almost perfect results close to 100%. In the KTR population, COVID pre-exposure almost doubles positive immunity test results from around 35–40% (unexposed) to 70–80% (exposed). This hybrid immunity result in the KTR population is remarkably better compared to a third vaccination attempt, where only about a third of non-responders will actually respond, adding up to response rates of 50–60% at best depending on the mRNA type used [14]. If vaccination circles are sequentially executed in a KTR population, always exposing non-responders to further vaccination, five circles need to be performed to reach positive seroconversion rates up to 80% as in hybrid immunity [15]. Our results are also consistent with another study by Gemander and coworkers, who demonstrated that hybrid immunity overcomes immune suppression, providing potent humoral and cellular immunity to SARS-CoV-2 in KTR independently of the usual risk factors associated with low vaccine responses in naïve KTRs [16].

While severe clinical outcomes were relatively rare in our patient population, other studies in normal populations already showed that individuals with lower IgG, IgA, and neutralizing antibody responses after vaccination had a significantly higher risk of reinfection and future Omicron infections [17]. The apparently increased effectiveness of hybrid immunity compared to repetitive vaccination in KTR may be explained by the broad repertoire of antigenic stimuli provided by the whole SARS-CoV-2 virus in comparison to the Spike protein of the vaccine. On the other hand, it is interesting that despite broad virus antigen exposition before vaccination, mRNA vaccines were not able to bring back immunological reactions in about 20% of these patients. Hereby, it needs to be considered that the numbers of pre-exposed KTR were relatively low and the time point of infection before vaccination was variable up to nine months [2].

Our second finding is that humoral immunity fading between two and nine months after vaccination is markedly attenuated by COVID-19 pre-exposure.

Using different measures (50% titer decrease or a strong decline qualification by RBD level categorization change—see Methods and Figures), RBD or IgG antibody decline between two and nine months after vaccination in COVID-19 unexposed participants was smallest in MP, intermediate in KTR, and highest in DP—a result which is consistent with other studies [18]. In COVID-19 pre-exposed study participants from all groups, a strong decline of RBD or IgG-Ab responses after vaccination was markedly reduced. At nine months, less than 10% of vaccinated and COVID-19 pre-exposed study participants (even in the DP group) showed a > 50% titer decline for RBD or IgG-Ab, while up to 63% (DP group) unexposed and vaccinated participants did. Hybrid immunity in the immunocompromised/immunosuppressed DP/KTR patient groups seems to overcome mechanisms leading to a strong decline of the immune response, achieving immunity stabilization to a similar level as observed in the MP group/the normal population, while also further improving immunity preservation in the MP group. Strong IgA antibody decline was less dependent on patient group or vaccine type but was markedly inhibited by COVID-19 pre-exposure in DP and KTR, while the results were less convincing in MP. Despite sample numbers for cellular immunity measurements via IGRA being lower, our results also suggest that COVID-19 pre-exposure has a similar stabilizing influence on cellular immunity as demonstrated for RBD and IgG-Ab responses. As discussed before, the broad repertoire of antigenic stimuli provided by the whole SARS-CoV-2 virus in comparison to the Spike protein of the vaccine and potentially the natural route of virus ingestion compared to vaccination may help to improve immunity preservation through hybrid immunity compared to vaccination alone.

Since immunity development in a normal population has been shown to be dependent on COVID-19 disease severity, we asked whether the clinical COVID-19 disease phenotype (symptomatic versus asymptomatic) may influence hybrid immunity development and/or preservation in immunocompromised DP, the group being large enough to explore this question [19,20]. Development of RBD antibody positivity after vaccination was not influenced by COVID-19 disease phenotype in DP. In contrast, the symptomatic clinical disease phenotype had an influence, being almost eight times stronger at preventing strong RBD-Ab decline in this vulnerable DP population compared to DP with asymptomatic COVID-19 pre-exposure that was just detected by antibody tests before vaccination. To our knowledge, this is the first study showing this association of symptomatic clinical disease with improved hybrid immunity preservation over time, which is especially pronounced in an otherwise fast-fading population of DP. While overall numbers of breakthrough infections were quite low during the study period, it is noticeable that hybrid immunity led to a predominance of asymptomatic breakthrough infections in all groups and to a lack of very severe cases in the vulnerable DP and KTR groups.

Even in this population of COVID-19 pre-exposed DP, the mRNA-1273 vaccine tended to be more successful in developing and preserving RBD antibody positivity when compared to BNT162b2mRNA. This difference was even prominent in the immunocompromised or immunosuppressed NoCOV groups of our study and is consistent with many other studies, where the mRNA-1273 vaccine type could be compared to BNT162b2mRNA [2,7].

Our study has several limitations related to its observational, non-randomized study characteristics and the lack of demographic matching between different cohorts. Although the study characteristics cannot be controlled for and may lead to hidden bias, demographic matching was accounted for in the multivariable analyses by including demographic factors as covariates. Financial resources limited the number of study participants in 26 out of 36 available dialysis centers of the Saxonian network. Study results were limited to patient groups with two closely applied vaccinations in order to be able to define a clear-cut starting and fading period, which is more difficult to execute in patients with a variable third and/or fourth vaccination pulse. At each time point, study participants were excluded from corresponding analyses for different reasons such as missed blood collections, withdrawal of consent, group switchers, etc. For logistical reasons, T-cell response could not be measured in all study participants. Using de novo NCP positivity for the exclusion of asymptomatic COVID-19 breakthrough infections from further analysis may underestimate the number of cases; however, this was performed for all participants [21].

## 5. Conclusions

In conclusion, these results indicate that SARS-CoV-2 virus pre-exposure, especially with clinically symptomatic disease (shown for the DP group), before vaccination not only elevates immunity rates and titers in immunocompromised/immunosuppressed DP/KTR to almost comparable levels with those of the normal population (MP) but also prevents immunity from fading for at least nine months after vaccination. Immune monitoring and vaccination efforts in vulnerable immunocompromised/immunosuppressed patient groups such as KTR and DP or even normal populations such as MP may be stretched in patients exposed to clinically overt hybrid immunity, especially when vaccinated with 1273-mRNA.

## Figures and Tables

**Table 3:** Positive immunity rates and titers at two months (T2) after the start of vaccination in different DIA-Vacc study groups (MP, DP, and KTR), separated by hybrid immunity (-COV) or its absence (-NoCOV).

Variable	Category	*MP-NoCOV*	*MP-COV*	*DP-NoCOV*	*DP-COV*	*KTR-NoCOV*	*KTR-COV*
**Humoral responses**							
IgG-Ab or IgA-Ab positive	n/total (%)	221/227 (97.4)	54/55 (98.2)	1282/1342 (95.5)	280/282 (99.3) ##	183/412 (44.4)	26/33 (78.8) ###
IgA-Ab positive	n/total (%)	221/227 (97.4)	54/55 (98.2)	1190/1342 (88.7)	273/282 (96.8) ###	164/412 (39.8)	25/33 (75.8) ###
IgA-Ab titer	Median interquartile range	8.7 (4.8–9)	8.5 (5.7–9)	6.9 (3–9)	9 (8.2–9) ###	0.7 (0.3–3.6)	9 (1.6–9) ###
IgG-Ab positive	n/total (%)	221/227 (97.4)	54/55 (98.2)	1270/1342 (94.6)	280/282 (99.3) ##	146/412 (35.4)	24/33 (72.7) ###
IgG-Ab titer	Median interquartile range	384 (384–384)	384 (384–384)	384 (384–384)	384 (384–384)	8.2 (3.2–154.8)	384 (4.2–384) ###
RBD-Ab positive	n/total (%)	188/190 (98.9)	48/50 (96)	1189/1290 (92.2)	278/281 (98.9) ###	118/296 (39.9)	21/29 (72.4) ##
RBD-Ab titer	Median interquartile range	99.4 (98.7–99.6)	99.6 (99.4–99.7) ###	97.3 (82.7–99.3)	99.7 (99.5–99.8) ###	12.9 (3.8–71.4)	99.5 (33.4–99.7) ###
**Cellular response**							
IGRA positive	n/total (%)	43/43 (100)	17/17 (100)	122/148 (82.4)	38/44 (86.4)	54/145 (37.2)	7/10 (70)
IGRA titer	Median interquartile range	2282 (860–2488)	2473 (2447–2487) #	808 (197–2470)	2465 (1029–2479) ##	29.4 (7–184)	357 (6–2346) #

Abbreviation for statistical significance (Chi-square test for antibody positivity rates and Mann–Whitney U test for antibody titers). Group comparisons between –Cov (COVID-19 infection prior to vaccination) and -NoCov (no COVID-19 infection prior to vaccination) in each group (MP/DP/KTR): # *p* < 0.05, ## *p* < 0.01, ### *p* < 0.001. MP = medical personnel; DP = dialysis patients; KTR = kidney transplant recipients.

**Table 4:** Success factors for RBD antibody positivity at two months (T2) after the start of vaccination for all participants: MP, DP, and KTR.

Multiple logistic regression analysis of RBD-Ab Positivity for All Participants			
*Success factor*	*Odds Ratio*	*95%CI*	*p-Value*
Age	0.985	[0.974,0.996]	0.010
Sex (Ref. = female)	1.151	[0.842, 1.573]	0.379
**COVID prior (Ref. = none)**	**4.511**	**[2.409, 8.447]**	**<0.001**
Vaccine type (Ref. = mRNA-1273)	0.437	[0.308, 0.621]	<0.001
DP (Ref. = MP)	0.264	[0.092, 0.757]	0.013
KTR (Ref. = MP)	0.012	[0.004, 0.034]	<0.001
Diabetes mellitus (Ref. = none)	1.118	[0.787, 1.587]	0.533
**Multiple logistic regression analysis of RBD-Ab Positivity for Medical Personnel**			
*Success factor*	*Odds Ratio*	*95%CI*	*p-value*
Age	0.904	[0.805, 1.015]	0.089
Sex (Ref. = female)	0.663	[0.06, 7.313]	0.737
COVID prior (Ref. = none)	0.167	[0.019, 1.447]	0.104
Vaccine type (Ref. = mRNA-1273)	1.457	[0.123, 17.32]	0.766
BMI	0.955	[0.794, 1.148]	0.622
**Multiple logistic regression analysis of RBD-Ab Positivity for Dialysis Patients**			
*Success factor*	*Odds Ratio*	*95%CI*	*p-value*
Age (years)	0.973	[0.956, 0.991]	0.003
Sex (Ref. = female)	1.33	[0.855, 2.07]	0.206
**COVID prior (Ref. = none)**	**6.615**	**[2.057, 21.268]**	**0.002**
Vaccine type (Ref. = mRNA-1273)	0.312	[0.193, 0.504]	<0.001
BMI	1.033	[0.988, 1.081]	0.148
IS drugs (Ref. = no drugs)	0.115	[0.058, 0.226]	<0.001
Time on dialysis	1.018	[0.979, 1.058]	0.378
Type of dialysis	2.769	[0.293, 26.155]	0.374
Hepatitis B vaccination failure	1.009	[0.588, 1.731]	0.873
Diabetes mellitus (Ref. = none)	0.835	[0.525, 1.329]	0.448
**Multiple logistic regression analysis of RBD-Ab Positivity for Kidney Transplant Recipients**			
*Success factor*	*Odds Ratio*	*95%CI*	*p-value*
Age	0.978	[0.958, 0.998]	0.036
Sex (Ref. = female)	1.094	[0.644, 1.858]	0.74
**COVID prior (Ref. = none)**	**5.239**	**[2.081, 13.191]**	**<0.001**
Vaccine type (Ref. = mRNA-1273)	0.687	[0.378, 1.251]	0.22
BMI	0.993	[0.943, 1.046]	0.786
IS number	0.452	[0.29, 0.704]	<0.001
Time after transplantation	1.063	[1.022, 1.106]	0.002
Hepatitis B vaccination failure	2.185	[0.879, 5.43]	0.092
Diabetes mellitus (Ref. = none)	1.424	[0.744, 2.725]	0.286

**Table 5:** Fifty percent antibody or IGRA fading rates (percentage) in comparison between two (T2) and nine months (T9) after vaccination start in different DIA-Vacc study groups (MP, DP, and KTR), separated by hybrid immunity (-COV) or not (-NoCOV) and vaccine type.

Variable	Group	Time	All	BNT162b2 mRNA	mRNA-1273
**Humoral responses**					
IgA-Ab decreasing (50%)	MP-NoCOV	T2 -> T9	124/202 (61.4%)	50/75 (66.7%)	74/127 (58.3%)
IgA-Ab decreasing (50%)	MP-COV	T2 -> T9	22/50 (44%) #	6/14 (42.9%)	16/36 (44.4%)
IgA-Ab decreasing (50%)	DP-NoCOV	T2 -> T9	504/711 (70.9%)	72/115 (62.6%)	432/596 (72.5%) *
IgA-Ab decreasing (50%)	DP-COV	T2 -> T9	44/176 (25%) ###	7/27 (25.9%)	37/149 (24.8%)
IgA-Ab decreasing (50%)	KTR-NoCOV	T2 -> T9	78/143 (54.5%)	13/28 (46.4%)	65/115 (56.5%)
IgA-Ab decreasing (50%)	KTR-COV	T2 -> T9	1/20 (5%) ###	1/6 (16.7%)	0/14 (0%)
IgG-Ab decreasing (50%)	MP-NoCOV	T2 -> T9	76/202 (37.6%)	40/75 (53.3%)	36/127 (28.3%) ***
IgG-Ab decreasing (50%)	MP-COV	T2 -> T9	5/50 (10%) ###	5/14 (35.7%)	0/36 (0%) ***
IgG-Ab decreasing (50%)	DP-NoCOV	T2 -> T9	450/711 (63.3%)	93/115 (80.9%)	357/596 (59.9%) ***
IgG-Ab decreasing (50%)	DP-COV	T2 -> T9	14/176 (8%) ###	4/27 (14.8%)	10/149 (6.7%)
IgG-Ab decreasing (50%)	KTR-NoCOV	T2 -> T9	63/143 (44.1%)	14/28 (50%)	49/115 (42.6%)
IgG-Ab decreasing (50%)	KTR-COV	T2 -> T9	0/20 (0%) ###	0/6 (0%)	0/14 (0%)
RBD-Ab decreasing (50%)	MP-NoCOV	T2 -> T9	30/169 (17.8%)	16/44 (36.4%)	14/125 (11.2%) ***
RBD-Ab decreasing (50%)	MP-COV	T2 -> T9	2/46 (4.3%) #	2/10 (20%)	0/36 (0%)
RBD-Ab decreasing (50%)	DP-NoCOV	T2 -> T9	381/730 (52.2%)	58/73 (79.5%)	323/657 (49.2%) ***
RBD-Ab decreasing (50%)	DP-COV	T2 -> T9	13/180 (7.2%) ###	4/20 (20%)	9/160 (5.6%)
RBD-Ab decreasing (50%)	KTR-NoCOV	T2 -> T9	51/132 (38.6%)	14/24 (58.3%)	37/108 (34.3%) *
RBD-Ab decreasing (50%)	KTR-COV	T2 -> T9	0/18 (0%) ##	0/4 (0%)	0/14 (0%)
**Interferon-γ release assay (IGRA) T-cellular response**					
IGRA decreasing (50%)	MP-NoCOV	T2 -> T9	22/39 (56.4%)	13/18 (72.2%)	9/21 (42.9%)
IGRA decreasing (50%)	MP-COV	T2 -> T9	3/12 (25%)	2/3 (66.7%)	1/9 (11.1%)
IGRA decreasing (50%)	DP-NoCOV	T2 -> T9	31/92 (33.7%)	13/33 (39.4%)	18/59 (30.5%)
IGRA decreasing (50%)	DP-COV	T2 -> T9	2/23 (8.7%) #	1/4 (25%)	1/19 (5.3%)
IGRA decreasing (50%)	KTR-NoCOV	T2 -> T9	17/32 (53.1%)	10/16 (62.5%)	7/16 (43.8%)
IGRA decreasing (50%)	KTR-COV	T2 -> T9	3/5 (60%)	3/4 (75%)	0/1 (0%)

Abbreviation for statistical significance (Chi-square test). Group comparisons between vaccine type (BNT162b2 versus mRNA1273): * *p* < 0.05, *** *p* < 0.001. Group comparisons between hybrid immunity due to COVID-19 infection prior to vaccination (-Cov) or not (-NoCov): # *p* < 0.05, ## *p* < 0.01, ### *p* < 0.001.

**Table 6:** Risk factors for RBD antibody fading between T2 and nine months (T9) based on the logistic regression model fitted for all participants, MP, DP, and KTR, respectively.

Multiple Logistic Regression Analysis of RBD-Ab Fading for All Patients			
**“Risk“ Factor**	***Odds Ratio***	**95%CI**	***p*** **-Value**
Age (years)	1.01	[1.001, 1.02]	0.036
Sex (Ref. = female)	1.268	[0.959, 1.676]	0.095
**COVID prior (Ref. = none)**	**0.084**	**[0.047, 0.151]**	**<0.001**
Vaccine type (Ref. = mRNA-1273)	2.141	[1.465, 3.128]	<0.001
Dialysis patients (Ref. = MP)	2.841	[1.762, 4.581]	<0.001
KT recipients (Ref. = MP)	0.934	[0.512, 1.705]	0.825
Diabetes mellitus (Ref. = none)	1.151	[0.855, 1.548]	0.353
**Multiple logistic regression analysis of RBD-Ab Fading for Medical Personnel**			
“Risk" factor	*Odds Ratio*	95%CI	*p*-value
Age (years)	1.059	[1.01, 1.11]	0.018
Sex (Ref. = female)	1.740	[0.623, 4.858]	0.29
**COVID prior (Ref. = none)**	**0.186**	**[0.038, 0.912]**	**0.038**
Vaccine type (Ref. = mRNA-1273)	7.717	[3.107, 19.168]	<0.001
BMI	0.89	[0.792, 0.999]	0.049
**Multiple logistic regression analysis of RBD-ab Fading for Dialysis Patients**			
“Risk“ factor	*Odds Ratio*	95%CI	*p*-value
Age (years)	1.002	[0.991, 1.013]	0.742
Sex (Ref. = female)	1.276	[0.922, 1.766]	0.142
**COVID prior (Ref. = none)**	**0.088**	**[0.046, 0.165]**	**<0.001**
Vaccine type (Ref. = mRNA-1273)	1.812	[1.117, 2.94]	0.016
BMI	0.985	[0.956, 1.014]	0.298
IS drugs (Ref. = no drugs)	0.804	[0.392, 1.651]	0.552
Time on dialysis	0.978	[0.952, 1.004]	0.10
Hep B vacc failure	1.173	[0.793, 1.735]	0.423
Diabetes mellitus (Ref. = none)	1.2	[0.856, 1.684]	0.29
**Multiple logistic regression analysis of RBD-Ab Fading for Kidney Transplant Recipients**			
“Risk“ factor	*Odds Ratio*	95%CI	*p*-value
Age (years)	1.042	[0.989, 1.098]	0.126
Sex (Ref. = female)	1.713	[0.54, 5.433]	0.361
COVID prior (Ref. = none)	0	N/a	0.991
Time after transplantation	1.029	[0.956, 1.107]	0.452
Vaccine type (Ref. = mRNA-1273)	1.059	[0.28, 4.006]	0.933
IS drugs number	0.754	[0.348, 1.632]	0.473
BMI	1.076	[0.963, 1.202]	0.196
Hep B vacc failure	0.811	[0.14, 4.702]	0.816
Diabetes mellitus (Ref. = none)	1.525	[0.459, 5.063]	0.491

**Table 7:** Success factors for RBD antibody positivity (T2) or fading between two (T2) and nine months (T9) after the start of vaccination for dialysis patients with hybrid immunity due to COVID-19 infection prior to vaccination. Multiple logistic regression analysis of RBD-Ab Positivity for DP with hybrid immunity.

Success Factor	*Odds Ratio*	95%CI	*p*-Value
Age (years)	0.947	[0.853, 1.05]	0.300
Sex (Ref. = female)	0	n/a	0.995
Prior COVID with Symptoms (Ref. = Asymptomatic)	2.359	[0.184, 30.204]	0.51
Vaccine type (Ref. = mRNA-1273)	0.106	[0.008, 1.461]	0.094
Diabetes mellitus (Ref. = none)	1.608	[0.133, 19.387]	0.708

**Table 8:** Success factors for RBD antibody positivity (T2) or fading between two (T2) and nine months (T9) after vaccination for dialysis patients with hybrid immunity due to COVID-19 infection prior to vaccination. Multiple logistic regression analysis of RBD-Ab Fading for DP with hybrid immunity.

“Risk” Factor	*Odds Ratio*	95%CI	*p*-Value
Age (years)	0.986	[0.94, 1.033]	0.548
Sex (Ref. = female)	0.78	[0.197, 3.08]	0.723
**Prior COVID with Symptoms (Ref. = Asymptomatic)**	**0.121**	**[0.015, 1.004]**	**0.05**
Vaccine type (Ref. = mRNA-1273)	3.602	[0.846, 15.338]	0.083
Hepatitis B vaccination failure	1.181	[0.208, 6.722]	0.851
Diabetes mellitus (Ref. = none)	1.554	[0.4, 6.043]	0.525

## Data Availability

The data can be shared up on request.

## Supplementary Materials

The following supporting information can be downloaded at: https://www.mdpi.com/article/10.3390/vaccines12070801/s1; Table S1A: Positive immunity rates at two months (T2) after vaccination start in different DIA-Vacc study groups (MP, DP, KTR), separated by hybrid immunity (-COV) or not (-NoCOV) and vaccine type (BNT162b2 mRNA/mRNA-1273); Table S1B: Positive immunity titers at two months (T2) after vaccination start in different DIA-Vacc study groups (MP, DP, KTR), separated by hybrid immunity (-COV) or not (-NoCOV) and vaccine type (BNT162b2mRNA/mRNA-1273; Table S2A: Immune response and 50% fading rates in comparison between two (T2) and nine (T9) months after vaccination start in different DIA-Vacc study groups (MP, DP, KTR), separated by hybrid immunity (-COV) or not (-NoCOV) and vaccine type; Table S2B: Antibody and IGRA titers at two (T2) and nine months (T9) after vaccination start in different DIA-Vacc study groups (MP, DP, KTR), separated by hybrid immunity (-COV) or not (-NoCOV); Table S2C: Antibody and IGRA titers two (T2) and nine months (T9) after vaccination in different DIA-Vacc study groups (MP, DP, KTR), separated by hybrid immunity (-COV) or not (-NoCOV) dependent on vaccine type; Table S3A: Interval categorization of RBD-Ab ranges of all participants; Table S3B: Interval categorization of RBD-Ab ranges of MP; Table S3C: Interval categorization of RBD-Ab ranges of DP; Table S3D: Interval categorization of RBD-Ab ranges of KTR; Table S3E: Interval categorization of RBD-Ab ranges of all DP with hybrid immunity; DIA-Vacc supplementary methods.
