# Supplementary material for: Hybrid Immunity Protects against Antibody Fading after SARS-CoV-2mRNA Vaccination in Kidney Transplant Recipients, Dialysis Patients, and Medical Personnel: 9 Months Data from the Prospective, Observational Dia-Vacc Study
Source: Vaccines (Basel). 2024 Jul 19;12(7):801. doi: 10.3390/vaccines12070801 (PMC11281450; doi:10.3390/vaccines12070801)
Supplement: Supplementary file 1 [file vaccines-12-00801-s001.zip › vaccines-3055962-supplementary.pdf]

The authors deleted the text before the words. The authors deleted the text before the words. Supplementary tables

Table S1A: Positive immunity rates at two months (T2) after vaccination start in different DIA-Vacc study groups (MP, DP, KTR), separated by hybrid immunity (-COV) or not (-NoCOV) and vaccine type (BNT162b2 mRNA / mRNA-1273)

Variable	Category	*MP-NoCOV*	*MP-COV*	*DP-NoCOV*	*DP-COV*	*KTR-NoCOV*	*KTR-COV*
**Humoral responses**							
**IgG-Ab or IgA-Ab positive**	n / total (%)	221 / 227 (97.4)	54 / 55 (98.2)	1282 / 1342 (95.5)	280 / 282 (99.3)##	183 / 412 (44.4)	26 / 33 (78.8)###
IgG-Ab or IgA-Ab positive – BNT162b2	n / total (%)	86 / 90 (95.6)	16 / 17 (94.1)	285 / 313 (91.1)	36 / 37 (97.3)	44 / 138 (31.9)	6 / 10 (60)
IgG-Ab or IgA-Ab positive – mRNA1273	n / total (%)	135 / 137 (98.5)	38 / 38 (100)	997 / 1029 (96.9)***	244 / 245 (99.6)	139 / 274 (50.7)***	20 / 23 (87)
**IgA-Ab positive**	n / total (%)	221 / 227 (97.4)	54 / 55 (98.2)	1190 / 1342 (88.7)	273 / 282 (96.8)###	164 / 412 (39.8)	25 / 33 (75.8)###
IgA-Ab positive – BNT162b2 mRNA	n / total (%)	86 / 90 (95.6)	16 / 17 (94.1)	227 / 313 (72.5)	30 / 37 (81.1)	36 / 138 (26.1)	5 / 10 (50)
IgA-Ab positive – mRNA1273	n / total (%)	135 / 137 (98.5)	38 / 38 (100)	963 / 1029 (93.6)***	243 / 245 (99.2)***	128 / 274 (46.7)***	20 / 23 (87)
**IgG-Ab positive**	n / total (%)	221 / 227 (97.4)	54 / 55 (98.2)	1270 / 1342 (94.6)	280 / 282 (99.3)##	146 / 412 (35.4)	24 / 33 (72.7)###
IgG-Ab positive – BNT162b2 mRNA	n / total (%)	86 / 90 (95.6)	16 / 17 (94.1)	278 / 313 (88.8)	36 / 37 (97.3)	32 / 138 (23.2)	6 / 10 (60)
IgG-Ab positive – mRNA1273	n / total (%)	135 / 137 (98.5)	38 / 38 (100)	992 / 1029 (96.4)***	244 / 245 (99.6)	114 / 274 (41.6)***	18 / 23 (78.3)
**RBD-Ab positive**	n / total (%)	188 / 190 (98.9)	48 / 50 (96)	1189 / 1290 (92.2)	278 / 281 (98.9)###	118 / 296 (39.9)	21 / 29 (72.4)##
RBD-Ab positive – BNT162b2 mRNA	n / total (%)	54 / 55 (98.2)	12 / 12 (100)	173 / 207 (83.6)	24 / 25 (96)	23 / 68 (33.8)	5 / 8 (62.5)
RBD-Ab positive – mRNA1273	n / total (%)	134 / 135 (99.3)	36 / 38 (94.7)	1016 / 1083 (93.8)***	254 / 256 (99.2)	95 / 228 (41.7)	16 / 21 (76.2)
**Cellular response**							
**IGRA positive**	**n / total (%)**	43 / 43 (100)	17 / 17 (100)	122 / 148 (82.4)	38 / 44 (86.4)	54 / 145 (37.2)	7 / 10 (70)
IGRA positive – BNT162b2 mRNA	n / total (%)	21 / 21 (100)	5 / 5 (100)	47 / 62 (75.8)	5 / 5 (100)	32 / 90 (35.6)	3 / 6 (50)
IGRA positive – mRNA1273	n / total (%)	22 / 22 (100)	12 / 12 (100)	75 / 86 (87.2)	33 / 39 (84.6)	22 / 55 (40)	4 / 4 (100)

Abbreviation for statistical significance (Chi-square test):
*group comparisons between vaccine type (BNT162b2 versus mRNA1273): * $p < 0.05$, ** $p < 0.01$, *** $p < 0.001$*
group comparisons between hybrid immunity due to COVID-19 infection prior vaccination (-Cov) or not (-NoCov): # $p < 0.05$, ## $p < 0.01$, ### $p < 0.001$

Table S1B: Positive immunity titres at two months (T2) after vaccination start in different DIA-Vacc study groups (MP, DP, KTR), separated by hybrid immunity (-COV) or not (-NoCOV) and vaccine type (BNT162b2mRNA / mRNA-1273)

Variable	Category	MP-NoCOV	MP-COV	DP-NoCOV	DP-COV	KTR-NoCOV	KTR-COV
**Humoral responses**							
**IgA-Ab titer**	Median interquartile range	8.7 (4.8 - 9)	8.5 (5.7 - 9)	6.9 (3 - 9)	9 (8.2 - 9)###	0.7 (0.3 - 3.6)	9 (1.6 - 9)###
IgA-Ab titer – BNT162b2 mRNA	Median interquartile range	4.7 (3 - 8)	5.8 (3.4 - 8.9)	2.6 (1 - 5.8)	3 (1.4 - 9)	0.5 (0.3 - 1.1)	0.6 (0.3 - 9)
IgA-Ab titer – mRNA1273	Median interquartile range	9 (8.3 - 9)***	8.9 (7.1 - 9)**	8.2 (4.3 - 9)***	9 (9 - 9)***	0.9 (0.3 - 5.2)***	9 (3.9 - 9)
**IgG-Ab titer**	Median interquartile range	384 (384 - 384)	384 (384 - 384)	384 (384 - 384)	384 (384 - 384)	8.2 (3.2 - 154.8)	384 (4.2 - 384)###
IgG-Ab titer – BNT162b2 mRNA	Median interquartile range	384 (384 - 384)	384 (384 - 384)	384 (128.4 - 384)	384 (384 - 384)	4.2 (3.2 - 28.8)	217.6 (3.2 - 384)
IgG-Ab titer – mRNA1273	Median interquartile range	384 (384 - 384)*	384 (384 - 384)	384 (384 - 384)***	384 (384 - 384)***	13.4 (3.2 - 297.1)***	384 (110.7 - 384)
**RBD-Ab titer**	Median interquartile range	99.4 (98.7 - 99.6)	99.6 (99.4 - 99.7)###	97.3 (82.7 - 99.3)	99.7 (99.5 - 99.8)###	12.9 (3.8 - 71.4)	99.5 (33.4 - 99.7)###
RBD-Ab titer – BNT162b2 mRNA	Median interquartile range	98.7 (97.3 - 99.4)	99.4 (98.3 - 99.5)	76.7 (51 - 94.3)	99.4 (97.2 - 99.6)	10.4 (4 - 58.5)	55.9 (6.4 - 99.5)
RBD-Ab titer – mRNA1273	Median interquartile range	99.4 (99.1 - 99.6)***	99.6 (99.5 - 99.8)*	98.3 (91 - 99.4)***	99.7 (99.5 - 99.8)***	12.9 (3.8 - 75.2)	99.6 (74.9 - 99.8)
**Cellular response**							
**IGRA titer**	Median interquartile range	2282 (860 - 2488)	2473 (2447 - 2487)#	808 (197 - 2470)	2465 (1029 - 2479)##	29.4 (7 - 184)	357 (6 - 2346)#
IGRA titer – BNT162b2 mRNA	Median interquartile range	1602 (740 - 2474)	2488 (2458 - 2492)	524 (123 - 2470)	1070 (802.3 - 2467.7)	26.6 (5 - 169)	6 (4 - 586)
IGRA titer – mRNA1273	Median interquartile range	2282 (938 - 2490)	2470 (2446 - 2484)	883 (355 - 2471)	2465 (1152 - 2483)	33.2 (10 - 213)	2276 (357 - 2346)

*Abbreviation for statistical significance (Mann-Whitney U test): group comparisons between vaccine type (BNT162b2 versus mRNA1273): * p < 0.05, ** p < 0.01, *** p < 0.001; group comparisons between hybrid immunity due to COVID-19 infection prior vaccination (-Cov) or not (-NoCov): # p < 0.05, ## p < 0.01, ### p < 0.001;*

Tables S1 describe the data relating to 2630 DiaVacc study participants (MP, DP, KTR) being evaluated for vaccination-dependent humoral or cellular positivity after two vaccinations at the two month time point (T2) being separated by hybrid immunity due to COVID-19 infection prior vaccination (-COV) or not (-NoCOV).

Positive immunity rates (Tables S1A) and titres (Table S1B) are demonstrated for all different subgroups together and separated by vaccine type either with 2x BNT162b2 mRNA or 2x mRNA-1273. MP = medical personnel; DP = dialysis patients; KTR = kidney transplant recipients

Participants experiencing asymptomatic or documented symptomatic** COVID-19 disease beyond T0 (also T1 – T4) were excluded from the immune response assessment in all tables and figures.*

Table S2A: Immune response and 50% fading rates in comparison between two (T2) and nine (T9) months after vaccination start in different DIA-Vacc study groups (MP, DP, KTR), separated by hybrid immunity (-COV) or not (-NoCOV) and vaccine type

Variable	Group	Time	All	BNT162b2 mRNA	mRNA-1273	p-value
**Humoral responses**						
IgG-Ab or IgA-Ab positive	MP-NoCOV	T2	215 / 220 (97.7%)	82 / 85 (96.5%)	133 / 135 (98.5%)	0.598
IgG-Ab or IgA-Ab positive	MP-NoCOV	T9	206 / 210 (98.1%)	74 / 77 (96.1%)	132 / 133 (99.2%)	0.279
IgG-Ab or IgA-Ab positive	MP-COV	T2	53 / 54 (98.1%)	15 / 16 (93.8%)	38 / 38 (100%)	0.652
IgG-Ab or IgA-Ab positive	MP-COV	T9	55 / 55 (100%)	15 / 15 (100%)	40 / 40 (100%)	
IgG-Ab or IgA-Ab positive	DP-NoCOV	T2	849 / 877 (96.8%)	156 / 164 (95.1%)	693 / 713 (97.2%)	0.265
IgG-Ab or IgA-Ab positive	DP-NoCOV	T9	706 / 830 (85.1%)	77 / 118 (65.3%)	629 / 712 (88.3%)	< 0.001
IgG-Ab or IgA-Ab positive	DP-COV	T2	203 / 204 (99.5%)	28 / 28 (100%)	175 / 176 (99.4%)	1
IgG-Ab or IgA-Ab positive	DP-COV	T9	191 / 194 (98.5%)	28 / 28 (100%)	163 / 166 (98.2%)	1
IgG-Ab or IgA-Ab positive	KTR-NoCOV	T2	136 / 172 (79.1%)	27 / 37 (73%)	109 / 135 (80.7%)	0.423
IgG-Ab or IgA-Ab positive	KTR-NoCOV	T9	117 / 160 (731 %)	21 / 30 (70%)	96 / 130 (73.8%)	0.842
IgG-Ab or IgA-Ab positive	KTR-COV	T2	19 / 24 (79.2%)	4 / 7 (57.1%)	15 / 17 (88.2%)	0.249
IgG-Ab or IgA-Ab positive	KTR-COV	T9	20 / 22 (90.9%)	4 / 6 (66.7%)	16 / 16 (100%)	0.112
IgA-Ab Spike S1 positive	MP-NoCOV	T2	215 / 220 (97.7%)	82 / 85 (96.5%)	133 / 135 (98.5%)	0.598
IgA-Ab Spike S1 positive	MP-NoCOV	T9	167 / 210 (79.5%)	49 / 77 (63.6%)	118 / 133 (88.7%)	< 0.001
IgA-Ab decreasing (50%)	MP-NoCOV	T2 -> T9	124 / 202 (61.4%)	50 / 75 (66.7%)	74 / 127 (58.3%)	0.301
IgA-Ab Spike S1 positive	MP-COV	T2	53 / 54 (98.1%)	15 / 16 (93.8%)	38 / 38 (100%)	0.652
IgA-Ab Spike S1 positive	MP-COV	T9	51 / 55 (92.7%)	13 / 15 (86.7%)	38 / 40 (95%)	0.633
IgA-Ab decreasing (50%)	MP-COV	T2 -> T9	22 / 50 (44%)	6 / 14 (42.9%)	16 / 36 (44.4%)	1
IgA-Ab Spike S1 positive	DP-NoCOV	T2	789 / 877 (90%)	122 / 164 (74.4%)	667 / 713 (93.5%)	< 0.001
IgA-Ab Spike S1 positive	DP-NoCOV	T9	524 / 830 (63.1%)	51 / 118 (43.2%)	473 / 712 (66.4%)	< 0.001

IgA-Ab decreasing (50%)	DP-NoCOV	T2 -> T9	504 / 711 (70.9%)	72 / 115 (62.6%)	432 / 596 (72.5%)	0.043
IgA-Ab Spike S1 positive	DP-COV	T2	199 / 204 (97.5%)	24 / 28 (85.7%)	175 / 176 (99.4%)	< 0.001
IgA-Ab Spike S1 positive	DP-COV	T9	183 / 194 (94.3%)	24 / 28 (85.7%)	159 / 166 (95.8%)	0.091
IgA-Ab decreasing (50%)	DP-COV	T2 -> T9	44 / 176 (25%)	7 / 27 (25.9%)	37 / 149 (24.8%)	1
IgA-Ab Spike S1 positive	KTR-NoCOV	T2	119 / 172 (69.2%)	20 / 37 (54.1%)	99 / 135 (73.3%)	0.04
IgA-Ab Spike S1 positive	KTR-NoCOV	T9	91 / 160 (56.9%)	15 / 30 (50%)	76 / 130 (58.5%)	0.523
IgA-Ab decreasing (50%)	KTR-NoCOV	T2 -> T9	78 / 143 (54.5%)	13 / 28 (46.4%)	65 / 115 (56.5%)	0.453
IgA-Ab Spike S1 positive	KTR-COV	T2	19 / 24 (79.2%)	4 / 7 (57.1%)	15 / 17 (88.2%)	0.249
IgA-Ab Spike S1 positive	KTR-COV	T9	18 / 22 (81.8%)	3 / 6 (50%)	15 / 16 (93.8%)	0.08
IgA-Ab decreasing (50%)	KTR-COV	T2 -> T9	1 / 20 (5%)	1 / 6 (16.7%)	0 / 14 (0%)	0.654
IgG-Ab Spike S1 positive	MP-NoCOV	T2	215 / 220 (97.7%)	82 / 85 (96.5%)	133 / 135 (98.5%)	0.598
IgG-Ab Spike S1 positive	MP-NoCOV	T9	206 / 210 (98.1%)	74 / 77 (96.1%)	132 / 133 (99.2%)	0.279
IgG-Ab decreasing (50%)	MP-NoCOV	T2 -> T9	76 / 202 (37.6%)	40 / 75 (53.3%)	36 / 127 (28.3%)	0.001
IgG-Ab Spike S1 positive	MP-COV	T2	53 / 54 (98.1%)	15 / 16 (93.8%)	38 / 38 (100%)	0.652
IgG-Ab Spike S1 positive	MP-COV	T9	54 / 55 (98.2%)	15 / 15 (100%)	39 / 40 (97.5%)	1
IgG-Ab decreasing (50%)	MP-COV	T2 -> T9	5 / 50 (10%)	5 / 14 (35.7%)	0 / 36 (0%)	0.001
IgG-Ab Spike S1 positive	DP-NoCOV	T2	845 / 877 (96.4%)	154 / 164 (93.9%)	691 / 713 (96.9%)	0.104
IgG-Ab Spike S1 positive	DP-NoCOV	T9	673 / 830 (81.1%)	66 / 118 (55.9%)	607 / 712 (85.3%)	< 0.001
IgG-Ab decreasing (50%)	DP-NoCOV	T2 -> T9	450 / 711 (63.3%)	93 / 115 (80.9%)	357 / 596 (59.9%)	< 0.001
IgG-Ab Spike S1 positive	DP-COV	T2	203 / 204 (99.5%)	28 / 28 (100%)	175 / 176 (99.4%)	1
IgG-Ab Spike S1 positive	DP-COV	T9	191 / 194 (98.5%)	28 / 28 (100%)	163 / 166 (98.2%)	1
IgG-Ab decreasing (50%)	DP-COV	T2 -> T9	14 / 176 (8%)	4 / 27 (14.8%)	10 / 149 (6.7%)	0.296
IgG-Ab Spike S1 positive	KTR-NoCOV	T2	125 / 172 (72.7%)	25 / 37 (67.6%)	100 / 135 (74.1%)	0.563
IgG-Ab Spike S1 positive	KTR-NoCOV	T9	111 / 160 (69.4%)	19 / 30 (63.3%)	92 / 130 (70.8%)	0.564

IgG-Ab decreasing (50%)	KTR-NoCOV	T2 -> T9	63 / 143 (44.1%)	14 / 28 (50%)	49 / 115 (42.6%)	0.621
IgG-Ab Spike S1 positive	KTR-COV	T2	19 / 24 (79.2%)	4 / 7 (57.1%)	15 / 17 (88.2%)	0.249
IgG-Ab Spike S1 positive	KTR-COV	T9	19 / 22 (86.4%)	4 / 6 (66.7%)	15 / 16 (93.8%)	0.342
IgG-Ab decreasing (50%)	KTR-COV	T2 -> T9	0 / 20 (0%)	0 / 6 (0%)	0 / 14 (0%)	
RBD-Ab Spike S1 positive	MP-NoCOV	T2	184 / 185 (99.5%)	52 / 52 (100%)	132 / 133 (99.2%)	1
RBD-Ab Spike S1 positive	MP-NoCOV	T9	190 / 209 (90.9%)	62 / 76 (81.6%)	128 / 133 (96.2%)	0.001
RBD-Ab decreasing (50%)	MP-NoCOV	T2 -> T9	30 / 169 (17.8%)	16 / 44 (36.4%)	14 / 125 (11.2%)	< 0.001
RBD-Ab Spike S1 positive	MP-COV	T2	47 / 49 (95.9%)	11 / 11 (100%)	36 / 38 (94.7%)	1
RBD-Ab Spike S1 positive	MP-COV	T9	52 / 55 (94.5%)	13 / 15 (86.7%)	39 / 40 (97.5%)	0.363
RBD-Ab decreasing (50%)	MP-COV	T2 -> T9	2 / 46 (4.3%)	2 / 10 (20%)	0 / 36 (0%)	0.062
RBD-Ab Spike S1 positive	DP-NoCOV	T2	818 / 881 (92.8%)	92 / 108 (85.2%)	726 / 773 (93.9%)	0.002
RBD-Ab Spike S1 positive	DP-NoCOV	T9	471 / 830 (56.7%)	29 / 118 (24.6%)	442 / 712 (62.1%)	< 0.001
RBD-Ab decreasing (50%)	DP-NoCOV	T2 -> T9	381 / 730 (52.2%)	58 / 73 (79.5%)	323 / 657 (49.2%)	< 0.001
RBD-Ab Spike S1 positive	DP-COV	T2	205 / 208 (98.6%)	20 / 21 (95.2%)	185 / 187 (98.9%)	0.704
RBD-Ab Spike S1 positive	DP-COV	T9	182 / 194 (93.8%)	24 / 28 (85.7%)	158 / 166 (95.2%)	0.134
RBD-Ab decreasing (50%)	DP-COV	T2 -> T9	13 / 180 (7.2%)	4 / 20 (20%)	9 / 160 (5.6%)	0.06
RBD-Ab Spike S1 positive	KTR-NoCOV	T2	105 / 153 (68.6%)	17 / 29 (58.6%)	88 / 124 (71%)	0.286
RBD-Ab Spike S1 positive	KTR-NoCOV	T9	69 / 160 (43.1%)	8 / 30 (26.7%)	61 / 130 (46.9%)	0.07
RBD-Ab decreasing (50%)	KTR-NoCOV	T2 -> T9	51 / 132 (38.6%)	14 / 24 (58.3%)	37 / 108 (34.3%)	0.05
RBD-Ab Spike S1 positive	KTR-COV	T2	18 / 21 (85.7%)	4 / 5 (80%)	14 / 16 (87.5%)	1
RBD-Ab Spike S1 positive	KTR-COV	T9	19 / 22 (86.4%)	4 / 6 (66.7%)	15 / 16 (93.8%)	0.342
RBD-Ab decreasing (50%)	KTR-COV	T2 -> T9	0 / 18 (0%)	0 / 4 (0%)	0 / 14 (0%)	
**Interferon-γ release assay (IGRA)**						

IGRA Spike S1 positive	MP-NoCOV	T2	43 / 43 (100%)	21 / 21 (100%)	22 / 22 (100%)	
IGRA Spike S1 positive	MP-NoCOV	T9	38 / 45 (84.4%)	15 / 21 (71.4%)	23 / 24 (95.8%)	0.066
IGRA decreasing (50%)	MP-NoCOV	T2 -> T9	22 / 39 (56.4%)	13 / 18 (72.2%)	9 / 21 (42.9%)	0.129
IGRA Spike S1 positive	MP-COV	T2	17 / 17 (100%)	5 / 5 (100%)	12 / 12 (100%)	
IGRA Spike S1 positive	MP-COV	T9	16 / 17 (94.1%)	6 / 7 (85.7%)	10 / 10 (100%)	0.853
IGRA decreasing (50%)	MP-COV	T2 -> T9	3 / 12 (25%)	2 / 3 (66.7%)	1 / 9 (11.1%)	0.248
IGRA Spike S1 positive	DP-NoCOV	T2	111 / 134 (82.8%)	40 / 53 (75.5%)	71 / 81 (87.7%)	0.111
IGRA Spike S1 positive	DP-NoCOV	T9	115 / 142 (81%)	26 / 37 (70.3%)	89 / 105 (84.8%)	0.091
IGRA decreasing (50%)	DP-NoCOV	T2 -> T9	31 / 92 (33.7%)	13 / 33 (39.4%)	18 / 59 (30.5%)	0.526
IGRA Spike S1 positive	DP-COV	T2	35 / 41 (85.4%)	5 / 5 (100%)	30 / 36 (83.3%)	0.754
IGRA Spike S1 positive	DP-COV	T9	34 / 38 (89.5%)	4 / 5 (80%)	30 / 33 (90.9%)	1
IGRA decreasing (50%)	DP-COV	T2 -> T9	2 / 23 (8.7%)	1 / 4 (25%)	1 / 19 (5.3%)	0.766
IGRA Spike S1 positive	KTR-NoCOV	T2	28 / 49 (57.1%)	18 / 30 (60%)	10 / 19 (52.6%)	0.832
IGRA Spike S1 positive	KTR-NoCOV	T9	17 / 46 (37%)	7 / 18 (38.9%)	10 / 28 (35.7%)	1
IGRA decreasing (50%)	KTR-NoCOV	T2 -> T9	17 / 32 (53.1%)	10 / 16 (62.5%)	7 / 16 (43.8%)	0.479
IGRA Spike S1 positive	KTR-COV	T2	6 / 8 (75%)	3 / 5 (60%)	3 / 3 (100%)	0.673
IGRA Spike S1 positive	KTR-COV	T9	6 / 8 (75%)	3 / 5 (60%)	3 / 3 (100%)	0.673
IGRA decreasing (50%)	KTR-COV	T2 -> T9	3 / 5 (60%)	3 / 4 (75%)	0 / 1 (0%)	0.819

Table S2B: Antibody and IGRA titres at two (T2) and nine months (T9) after vaccination start in different DIA-Vacc study groups (MP, DP, KTR), separated by hybrid immunity (-COV) or not (-NoCOV)

Variable	Group	Category	Two months (T2)	Nine months (T4)	p-value
**Humoral responses**					
IgA-Ab Spike S1	MP-NoCOV	Median (interquartile range)	8.9 (5.1 - 9)	2.7 (1.3 - 5.3)	< 0.001
IgA-Ab Spike S1	MP-COV	Median (interquartile range)	8.3 (5.7 - 9)	3.7 (2.3 - 8)	< 0.001
IgA-Ab Spike S1	DP-NoCOV	Median (interquartile range)	7.1 (3.2 - 9)	1.7 (0.8 - 4.1)	< 0.001
IgA-Ab Spike S1	DP-COV	Median (interquartile range)	9 (8.1 - 9)	6.6 (3.8 - 8)	< 0.001
IgA-Ab Spike S1	KTR-NoCOV	Median (interquartile range)	3.6 (0.7 - 8.8)	1.4 (0.5 - 3.7)	< 0.001
IgA-Ab Spike S1	KTR-COV	Median (interquartile range)	8.9 (5.1 - 9)	2.7 (1.3 - 5.3)	< 0.001
IgG-Ab Spike S1	MP-NoCOV	Median (interquartile range)	9 (3.9 - 9)	8 (2.4 - 8)	0.001
IgG-Ab Spike S1	MP-COV	Median (interquartile range)	384 (384 - 384)	232.6 (151.2 - 357.7)	< 0.001
IgG-Ab Spike S1	DP-NoCOV	Median (interquartile range)	384 (384 - 384)	384 (300.6 - 384)	0.016
IgG-Ab Spike S1	DP-COV	Median (interquartile range)	384 (384 - 384)	124.4 (44.8 - 257.5)	< 0.001
IgG-Ab Spike S1	KTR-NoCOV	Median (interquartile range)	384 (384 - 384)	384 (384 - 384)	< 0.001
IgG-Ab Spike S1	KTR-COV	Median (interquartile range)	204.5 (17.7 - 384)	82.4 (25.1 - 245.9)	< 0.001
RBD-IgG	MP-NoCOV	Median (interquartile range)	99.4 (98.8 - 99.6)	74.4 (55.1 - 89.4)	< 0.001
RBD-IgG	MP-COV	Median (interquartile range)	99.6 (99.4 - 99.7)	97.5 (87.3 - 99.1)	< 0.001
RBD-IgG	DP-NoCOV	Median (interquartile range)	97.5 (85 - 99.3)	42.2 (18.6 - 74.2)	< 0.001
RBD-IgG	DP-COV	Median (interquartile range)	99.7 (99.5 - 99.8)	99.1 (94.9 - 99.7)	< 0.001
RBD-IgG	KTR-NoCOV	Median (interquartile range)	61.9 (19 - 92)	29.8 (10.1 - 66.8)	< 0.001
RBD-IgG	KTR-COV	Median (interquartile range)	99.6 (95.7 - 99.8)	98.8 (66 - 99.8)	0.022
**IGRA– T-cellular**					

IGRA	MP-NoCOV	Median (interquartile range)	2282.1 (860 - 2487.7)	664.4 (137.5 - 1477.4)	< 0.001
IGRA	MP-COV	Median (interquartile range)	2472.9 (2447.3 - 2487)	1777.6 (446.8 - 2466.1)	0.009
IGRA	DP-NoCOV	Median (interquartile range)	831.1 (213.4 - 2469.6)	555.5 (131 - 1755)	< 0.001
IGRA	DP-COV	Median (interquartile range)	2465.1 (1029.1 - 2478.6)	2412.4 (883.7 - 2475.8)	0.687
IGRA	KTR-NoCOV	Median (interquartile range)	133.9 (14.5 - 392)	59.5 (11.2 - 153.7)	0.003
IGRA	KTR-COV	Median (interquartile range)	356.7 (6.3 - 2275.6)	160.7 (0 - 1046.4)	0.062

Table S2C: Antibody and IGRA titres two (T2) and nine months (T9) after vaccination in different DIA-Vacc study groups (MP, DP, KTR), separated by hybrid immunity (-COV) or not (-NoCOV) dependent on vaccine type

Variable	Group	Time point	Category	BNT162b2 mRNA	mRNA-1273	p-value (Mann–Whitney U test)
**Humoral responses**						
IgA-Ab Spike S1	MP-NoCOV	T2	Median (interquartile range)	4.8 (3 - 8.1)	9 (8.2 - 9)	< 0.001
IgA-Ab Spike S1	MP-NoCOV	T9	Median (interquartile range)	1.6 (0.8 - 2.9)	3.6 (1.7 - 6.1)	< 0.001
IgA-Ab Spike S1	MP-COV	T2	Median (interquartile range)	5.3 (3.3 - 8.3)	8.9 (7.1 - 9)	0.003
IgA-Ab Spike S1	MP-COV	T9	Median (interquartile range)	3.1 (1.3 - 5.2)	4 (2.5 - 8)	0.199
IgA-Ab Spike S1	DP-NoCOV	T2	Median (interquartile range)	2.6 (1.1 - 5.8)	8.2 (4 - 9)	< 0.001
IgA-Ab Spike S1	DP-NoCOV	T9	Median (interquartile range)	0.9 (0.5 - 1.9)	1.9 (0.8 - 4.2)	< 0.001
IgA-Ab Spike S1	DP-COV	T2	Median (interquartile range)	4.1 (1.4 - 9)	9 (9 - 9)	< 0.001
IgA-Ab Spike S1	DP-COV	T9	Median (interquartile range)	7.1 (2.5 - 8)	6.6 (4 - 8)	0.929
IgA-Ab Spike S1	KTR-NoCOV	T2	Median (interquartile range)	1.3 (0.5 - 4.3)	4 (0.9 - 9)	0.002
IgA-Ab Spike S1	KTR-NoCOV	T9	Median (interquartile range)	0.7 (0.4 - 2.6)	1.4 (0.6 - 4)	0.151
IgA-Ab Spike S1	KTR-COV	T2	Median (interquartile range)	4 (0.3 - 9)	9 (8.2 - 9)	0.144
IgA-Ab Spike S1	KTR-COV	T9	Median (interquartile range)	0.9 (0.1 - 8)	8 (5.6 - 8)	0.171
IgG-Ab Spike S1	MP-NoCOV	T2	Median (interquartile range)	384 (384 - 384)	384 (384 - 384)	0.039
IgG-Ab Spike S1	MP-NoCOV	T9	Median (interquartile range)	184 (108.1 - 265.5)	257.8 (181.7 - 384)	< 0.001
IgG-Ab Spike S1	MP-COV	T2	Median (interquartile range)	384 (384 - 384)	384 (384 - 384)	0.159
IgG-Ab Spike S1	MP-COV	T9	Median (interquartile range)	196.1 (117.6 - 384)	384 (384 - 384)	< 0.001
IgG-Ab Spike S1	DP-NoCOV	T2	Median (interquartile range)	384 (143.6 - 384)	384 (384 - 384)	< 0.001
IgG-Ab Spike S1	DP-NoCOV	T9	Median (interquartile range)	42.8 (16.3 - 114.5)	141.4 (56.7 - 267.1)	< 0.001

IgG-Ab Spike S1	DP-COV	T2	Median (interquartile range)	384 (384 - 384)	384 (384 - 384)	< 0.001
IgG-Ab Spike S1	DP-COV	T9	Median (interquartile range)	384 (292.7 - 384)	384 (384 - 384)	0.059
IgG-Ab Spike S1	KTR-NoCOV	T2	Median (interquartile range)	90.1 (5.7 - 380.8)	267.6 (23.2 - 384)	0.022
IgG-Ab Spike S1	KTR-NoCOV	T9	Median (interquartile range)	53 (12 - 228.4)	91.2 (28.7 - 255.8)	0.289
IgG-Ab Spike S1	KTR-COV	T2	Median (interquartile range)	217.6 (3.2 - 384)	384 (384 - 384)	0.067
IgG-Ab Spike S1	KTR-COV	T9	Median (interquartile range)	133.5 (3.2 - 384)	384 (352 - 384)	0.141
RBD-IgG-Ab	MP-NoCOV	T2	Median (interquartile range)	98.7 (97.3 - 99.4)	99.4 (99.1 - 99.6)	< 0.001
RBD-IgG-Ab	MP-NoCOV	T9	Median (interquartile range)	57.2 (40 - 76.4)	80.1 (66.8 - 90.9)	< 0.001
RBD-IgG-Ab	MP-COV	T2	Median (interquartile range)	99.4 (98.3 - 99.6)	99.6 (99.5 - 99.8)	0.018
RBD-IgG-Ab	MP-COV	T9	Median (interquartile range)	60.8 (52.4 - 97.1)	98.1 (95.1 - 99.2)	< 0.001
RBD-IgG-Ab	DP-NoCOV	T2	Median (interquartile range)	85.4 (54.4 - 95.1)	98.2 (90 - 99.4)	< 0.001
RBD-IgG-Ab	DP-NoCOV	T9	Median (interquartile range)	17.3 (4.5 - 33.3)	48.2 (23 - 75.9)	< 0.001
RBD-IgG-Ab	DP-COV	T2	Median (interquartile range)	99.5 (98.8 - 99.6)	99.7 (99.6 - 99.8)	< 0.001
RBD-IgG-Ab	DP-COV	T9	Median (interquartile range)	96.4 (71 - 99.6)	99.2 (95.7 - 99.7)	0.043
RBD-IgG-Ab	KTR-NoCOV	T2	Median (interquartile range)	41.5 (19 - 77.6)	68.2 (18.7 - 95)	0.165
RBD-IgG-Ab	KTR-NoCOV	T9	Median (interquartile range)	14.5 (2.4 - 55.4)	33.5 (13.2 - 69)	0.024
RBD-IgG-Ab	KTR-COV	T2	Median (interquartile range)	99.5 (55.9 - 99.6)	99.6 (95.7 - 99.8)	0.2
RBD-IgG-Ab	KTR-COV	T9	Median (interquartile range)	45.6 (0 - 99.6)	98.8 (78.3 - 99.8)	0.357
**IGRA– T-cellular**						
IGRA	MP-NoCOV	T2	Median (interquartile range)	1602.3 (740.2 - 2474.5)	2282.1 (937.6 - 2490.3)	0.285
IGRA	MP-NoCOV	T9	Median (interquartile range)	186.2 (73.6 - 664.4)	1105.6 (496.4 - 1782.9)	0.003
IGRA	MP-COV	T2	Median (interquartile range)	2487.8 (2458.2 - 2491.7)	2470.4 (2446.1 - 2483.6)	0.442
IGRA	MP-COV	T9	Median (interquartile range)	375.6 (116.8 - 1005.4)	2322.8 (1851.9 - 2472.8)	< 0.001

IGRA	DP-NoCOV	T2	Median (interquartile range)	524.4 (139.5 - 2469.6)	1008.6 (384.5 - 2459.7)	0.131
IGRA	DP-NoCOV	T9	Median (interquartile range)	231.7 (70.5 - 1503.4)	603.1 (220.4 - 2206.6)	0.108
IGRA	DP-COV	T2	Median (interquartile range)	1070 (802.3 - 2467.7)	2465.1 (1029.1 - 2478.6)	0.563
IGRA	DP-COV	T9	Median (interquartile range)	883.7 (408.2 - 1596.9)	2430.1 (1021.1 - 2475.8)	0.187
IGRA	KTR-NoCOV	T2	Median (interquartile range)	133.9 (14.5 - 326.4)	106.8 (14.3 - 879)	0.911
IGRA	KTR-NoCOV	T9	Median (interquartile range)	62.8 (11.2 - 146.1)	39 (10.7 - 181.9)	0.866
IGRA	KTR-COV	T2	Median (interquartile range)	234.2 (6.3 - 586.2)	2275.6 (356.7 - 2346)	0.393
IGRA	KTR-COV	T9	Median (interquartile range)	143 (0 - 1046.4)	274.4 (160.7 - 2402.9)	0.368

Tables S2 describe the data relating to 1796 DiaVacc study participants (MP, DP, KTR) being evaluated for vaccination-dependent humoral or cellular positivity after two vaccinations at the nine month (T9) in comparison to the two month time point (T2) being separated by hybrid immunity due to COVID-19 infection prior vaccination (-COV) or not (-NoCOV) and focusing on immunity fading.

Positive immunity rates (Table S2A) on T9 in comparison with T2 are demonstrated for all different subgroups together or separated in participants being vaccinated with 2x BNT162b2 mRNA or 2x mRNA-1273. To assess immunity fading of antibody or IGRA titers between T2 and T4, a 50% margin was categorized as decreased (< 50%). Table S2B compares the average titer levels (median/interquartile range) on T9 with T2 (different columns) for the different anti-Spike S1 IgA, IgG, RBD-IgG antibodies as well as for cellular immunity via IGRA measurements. Table S2C compares the average titer levels (median/interquartile range) on T2 and T9 (different rows) for the different anti-Spike S1 IgG, RBD-IgG antibodies as well as for cellular immunity via IGRA measurements separated for the basic vaccine type (2x BNT162b2mRNA or 2x 1273-mRNA in different columns). Medical personnel (MP), dialysis patients (DP) or kidney transplant recipients KTR) were evaluated.
Participants experiencing asymptomatic or documented symptomatic** COVID-19 disease beyond T0 were excluded from the immune response assessment in all tables.*

Table S3A: Interval categorization of RBD-Ab ranges of all participants

RBD Level	Interval [%]	N of Participants at T2	N of Participants at T9
**0**	RBD < 35	120	487
**1**	35 ≤ RBD < 50	45	155
**2**	50 ≤ RBD < 65	63	161
**3**	65 ≤ RBD < 80	85	159
**4**	RBD ≥ 80	1184	508

Table S3B: Interval categorization of RBD-Ab ranges of MP

RBD Level	Interval [%]	N of Participants at T2	N of Participants at T9
**0**	RBD < 35	3	22
**1**	35 ≤ RBD < 50	0	28
**2**	50 ≤ RBD < 65	0	39
**3**	65 ≤ RBD < 80	2	47
**4**	RBD ≥ 80	229	128

Table S3C: Interval categorization of RBD-Ab ranges of DP

RBD Level	Interval [%]	N of Participants at T2	N of Participants at T9
**0**	RBD < 35	66	371
**1**	35 ≤ RBD < 50	26	110
**2**	50 ≤ RBD < 65	51	112
**3**	65 ≤ RBD < 80	61	93
**4**	RBD ≥ 80	885	338

Table S3D: Interval categorization of RBD-Ab ranges of KTR

RBD Level	Interval [%]	N of Participants at T2	N of Participants at T9
**0**	RBD < 35	51	94
**1**	35 ≤ RBD < 50	19	17
**2**	50 ≤ RBD < 65	12	10
**3**	65 ≤ RBD < 80	22	19
**4**	RBD ≥ 80	70	42

Table S3E: Interval categorization of RBD-Ab ranges of all DP with hybrid immunity

RBD Level	Interval [%]	N of Participants at T2	N of Participants at T9
**0**	RBD < 35	3	12
**1**	35 ≤ RBD < 50	1	3
**2**	50 ≤ RBD < 65	1	6
**3**	65 ≤ RBD < 80	1	9
**4**	RBD ≥ 80	202	164

Tables S3A-E show the overall numbers of study participants being categorized in the interval classification that was used for multivariable analysis of risk factors for a "strong RBD-ab decline". *Tables S3A-D summarize the absolute frequencies in the corresponding populations/groups including COVID-19 infected and nonexposed participants prior vaccination. Table S3E summarizes the absolute frequencies in the DP group with hybrid immunity only.*
